# Improved CRISPR genome editing using small highly active and specific engineered RNA-guided nucleases

**DOI:** 10.1038/s41467-021-24454-5

**Published:** 2021-07-09

**Authors:** Moritz J. Schmidt, Ashish Gupta, Christien Bednarski, Stefanie Gehrig-Giannini, Florian Richter, Christian Pitzler, Michael Gamalinda, Christina Galonska, Ryo Takeuchi, Kui Wang, Caroline Reiss, Kerstin Dehne, Michael J. Lukason, Akiko Noma, Cindy Park-Windhol, Mariacarmela Allocca, Albena Kantardzhieva, Shailendra Sane, Karolina Kosakowska, Brian Cafferty, Jan Tebbe, Sarah J. Spencer, Scott Munzer, Christopher J. Cheng, Abraham Scaria, Andrew M. Scharenberg, André Cohnen, Wayne M. Coco

**Affiliations:** 1 Bayer AG, Leverkusen, Germany; 2 Casebia Therapeutics LLC, Cambridge, MA USA; 3 CRISPR Therapeutics INC, Cambridge, MA USA

**Keywords:** Gene therapy, CRISPR-Cas9 genome editing

## Abstract

*Streptococcus pyogenes* (Spy) Cas9 has potential as a component of gene therapeutics for incurable diseases. One of its limitations is its large size, which impedes its formulation and delivery in therapeutic applications. Smaller Cas9s are an alternative, but lack robust activity or specificity and frequently recognize longer PAMs. Here, we investigated four uncharacterized, smaller Cas9s and found three employing a “GG” dinucleotide PAM similar to SpyCas9. Protein engineering generated synthetic RNA-guided nucleases (sRGNs) with editing efficiencies and specificities exceeding even SpyCas9 in vitro and in human cell lines on disease-relevant targets. sRGN mRNA lipid nanoparticles displayed manufacturing advantages and high in vivo editing efficiency in the mouse liver. Finally, sRGNs, but not SpyCas9, could be packaged into all-in-one AAV particles with a gRNA and effected robust in vivo editing of non-human primate (NHP) retina photoreceptors. Human gene therapy efforts are expected to benefit from these improved alternatives to existing CRISPR nucleases.

## Abstract (web summary)

Small CRISPR Cas9 proteins have potential in gene therapies but generally have poor editing efficiency or specificity and often recognize sub-optimal PAM sequences. Here the authors characterise four small nucleases and use protein engineering to create effective in vivo editors.

## Introduction

CRISPR systems evolved as a bacterial adaptive immune system in which resistance to phage infection is mediated by a nuclease (e.g., Cas9) that cleaves phage DNA. By exploiting the base-pairing potential of a guide RNA (gRNA), Cas9 targets a corresponding genomic locus for cleavage. A short motif on the targeted DNA, termed the protospacer adjacent motif (PAM), is necessary for Cas9 activity^1–4^. CRISPR-Cas9 has been harnessed as a versatile tool for directed genome editing^5–7^ and, with the above constraints, allows the targeting in principle of any given chromosomal region of interest.

*Streptococcus pyogenes* Cas9 (SpyCas9), the most common enzyme used in genome-editing applications, is a large nuclease of 1368 amino acid residues^5^. The advantages of SpyCas9 include its short, 5′-NGG-3′ PAM and very high average editing efficiency. Despite concerns about its specificity profile in some applications, the listed features have led SpyCas9 to be explored among the CRISPR nucleases of choice for clinical gene therapy applications^8^.

Effective delivery of CRISPR-Cas systems to targeted cells and tissues is crucial for successful in vivo genome editing. For this purpose, recombinant adeno-associated virus (rAAV) vectors and lipid-nanoparticles (LNPs) are among the most prevalent and promising technologies^9^. Because packaging into rAAV vectors is limited to ~5 kb, Cas9 proteins smaller than SpyCas9 are desirable to enable packaging of DNA encoding both Cas9 and sgRNA into one rAAV (“all-in-one-AAV”) particle. This limitation is exacerbated for the larger multidomain-Cas-nuclease-based systems for base editing, prime editing, or CRISPRi/a^10–14^. Beyond rAAVs, smaller nucleases can also facilitate formulation and mRNA manufacturing for LNPs^15^. Smaller Cas9 proteins are thus of keen interest in the field.

The best-characterized smaller Cas9 are from *Staphylococcus aureus* (SauCas9, 1053 amino acid residues)^16^ and *Campylobacter jejuni* (CjCas9, 984 amino residues)^17^. However, both recognize longer PAMs, 5′-NNGRRT-3′ for SauCas9 (R = A or G) and 5′-NNNNRYAC-3′ for CjCas9 (Y = C or T), which reduces the number of uniquely addressable target sites in the genome, in comparison to the NGG SpyCas9 PAM. First reports indicate that SauCas9 specificity is similar to SpyCas9^18,19^.

Protein engineering and directed protein evolution have been successfully applied to improve a wide range of properties of many therapeutic and nontherapeutic proteins, including SpyCas9^20–26^. Gene family DNA shuffling is a powerful protein optimization approach that leverages sequence diversity from homologous genes by randomly swapping gene fragments or polymorphisms to generate screenable gene-variant libraries^20,22,23^. This allows a myriad of perturbations of protein structure and function, while simultaneously maintaining a relatively high fraction of functional clones in the libraries^20–23^. The compounded effects of the perturbations in each clone can have minor to major effects on the encoded protein’s phenotype. Screening such a library can thus generate improvements involving combinations of a large number of simultaneous mutations, which are poorly accessible by other engineering approaches^21,22^.

Here, we apply gene family shuffling to four small Cas9 nucleases (~1050 amino acid residues). Our aim was to generate short, but highly active and specific, synthetic RNA-guided nucleases (sRGNs, pronounced “surgeons”) that recognize the favorable “GG” di-nucleotide PAM. Our resulting sRGNs displayed higher specificity and activity than SpyCas9 in human cell lines and robust in vivo editing in mice and nonhuman primates (NHP) when formulated as mRNA into LNPs, as well as when packaged as DNA into a single rAAV vector, thus making them well-suited candidates for gene therapy applications.

## Results

### Characterization of four related small Cas9 nucleases

**Fig. 1:**
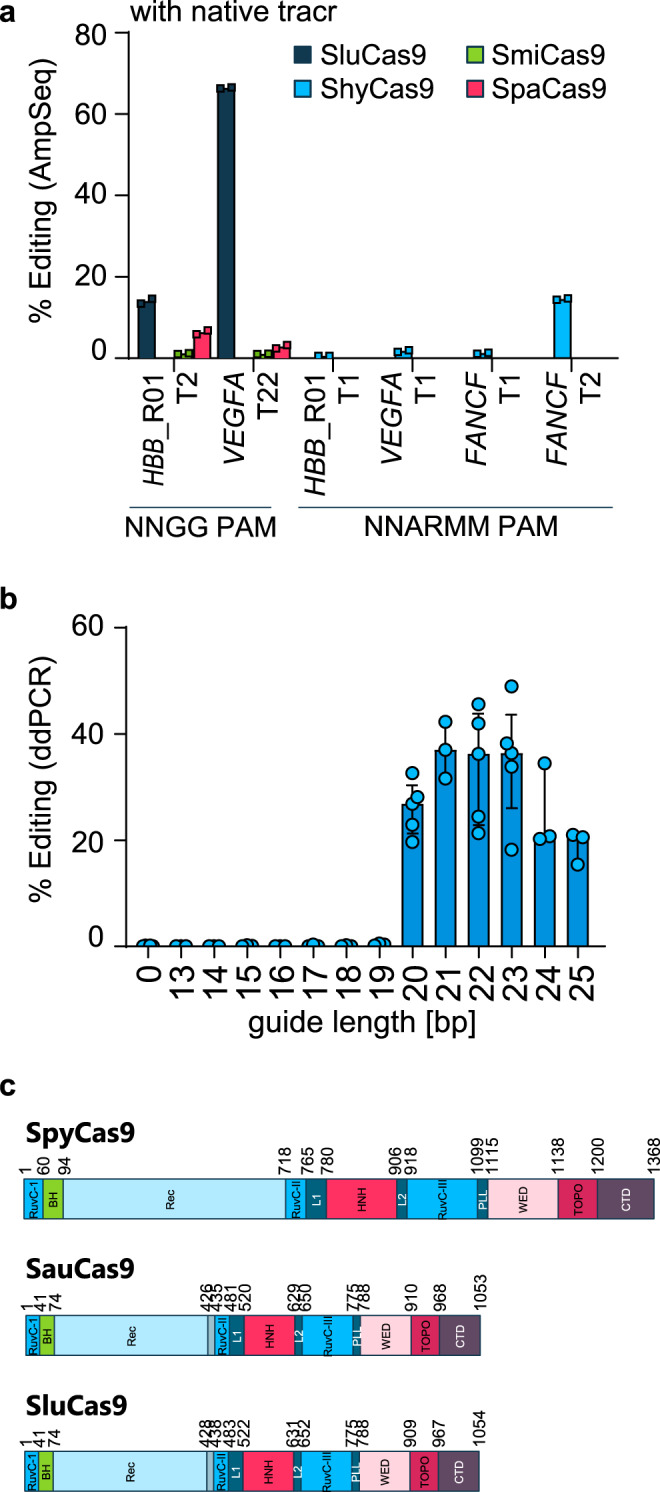
Genome editing and domain architecture of four *Staphylococcus* Cas9s. **a** Activity of wild-type Cas9s on HEK293T loci assayed by amplicon sequencing. Cas9s were tested as RNPs with their respective native tracr sequences and delivered via nucleofection. Spa, Smi, and Slu were tested on two targets with 5′-NNGG-3′ PAM (guide_87 and guide_102 targeting the *HBB*_R01_T2 and *VEGFA*_T22 loci, respectively). Because of the distinct PAM for ShyCas9, the corresponding experiments with ShyCas9 required a different set of targets in these loci containing a 5′-NNARMM-3′ PAM (guide_1-4 targeting the *HBB*_R01_T1, *VEGFA*_T1 and *FANCF*_T1, and *FANCF*_T2 loci). Data are presented as the mean of *n* = 2 independent biological replicates. Editing values were background subtracted. **b** Effect of guide length on the efficiency of SluCas9 editing. To assess the optimal protospacer length for SluCas9, a single *HBB* site (R01_T2) was targeted by synthetic sgRNAs with protospacer lengths varying from 13 to 25 nt (guide_267, 268, and guide_80-90) using RNP nucleofection. The editing efficiency was measured using ddPCR. For negative controls, the nuclease was nucleofected in the absence of sgRNA. Shown are individual measurements with the median as bars plus interquartile range, *n* = 3 for length 13–19, 21, 24, and 25, *n* = 5 for length 20, 22, and 23 independent biological replicates. **c** Domain architecture schematic for SpyCas9^62^ and SauCas9^63^, and putative domain architecture for SluCas9 based on an alignment with SauCas9. Source data of 1a and 1b are provided in the source data file.

We examined four related, previously uncharacterized Cas9s from *Staphylococcus hyicus* (Shy), *Staphylococcus lugdunensis* (Slu), *Staphylococcus microti* (Smi), and *Staphylococcus pasteuri* (Spa), and found three recognized a favorable “NNGG” PAM (Supplementary Fig. 1). Among these, only SluCas9 displayed prominent genome-editing activity using ribonucleoprotein particles (RNP) directed to the *HBB* or the *VEGFA* locus in mammalian cell lines (Fig. 1a). The tracrRNA for all four parents were similar; however, upon assessing each of the four nucleases for activity with the SluCas9 tracrRNA, we observed increased genome editing only for ShyCas9 (Supplementary Fig. 2). The optimal guide length for SluCas9 was 21–23 nucleotides (Fig. 1b), similar to previous reports on SauCas9^16^. Homology mapping to SpyCas9 and SauCas9 yielded predicted protein domains of the nucleases (Fig. 1c), facilitating the generation of nickases and inactive (“dead”) enzymes (Supplementary Fig. 1e).

### Protein engineering and characterization of improved nucleases

**Fig. 2:**
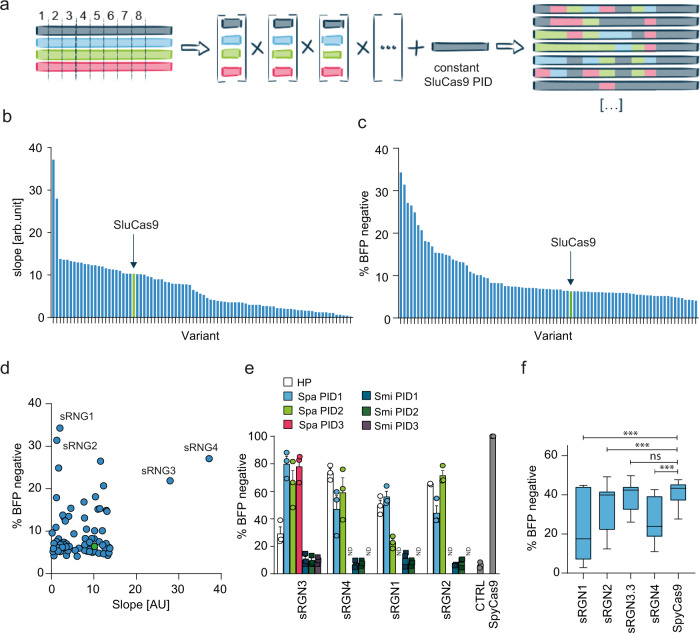
Gene family DNA shuffling, screening, and confirmation of engineered, chimeric hits. **a** Schematic of the employed gene family DNA shuffling approach. Parental Cas9 gene fragments were generated with termini at 8 or 11 positions of high sequence identity and reassembled to yield gene family DNA-shuffled libraries containing synthetic RNA-guided nuclease (sRGN) genes with 8 or 11 fixed cross-over points. Each shuffled library member possessed a constant SluCas9 PI-domain (PID) to maintain NNGG PAM recognition. **b** Activity preselection using a “live/dead” bacterial survival assay, followed by a cell free-cleavage fluorescence polarization (FP) assay on the *VEGFA*_T2 target yielded 165 active chimeric nucleases. Slope values correlate with enzyme activity. A representative experiment is shown (*n* = 1), arb.unit = arbitrary units. Green bar = SluCas9 control. Source data are presented in the source data file. **c** HEK293T cells containing a genome-integrated *BFP* gene were transfected with plasmids encoding each of the 165 sRGN variants. The editing efficiency was assessed by the loss of BFP fluorescence. The gating strategy is shown in Supplementary Fig. 10. Data are presented as the mean of *n* = 2 independent biological replicates. Green bar = SluCas9 control. **d** Correlation of FP and BFP disruption data shown in **b** and **c** identified two top hits for each assay; sRGN1-4 were selected for further analyses, green dot = SluCas9. **e** SpaPID1-3 and SmiPID1-3 denote replacement of three alternative stretches comprising the constant Slu PID in the shuffled sRGN screening hits with corresponding parts of either SpaCas9 or SmiCas9. HP (hit PID) denotes the respective hit nuclease with its original PID. The values are normalized to SpyCas9. Data represent *n* = 3 independent biological replicates with mean ± SEM. *ND* = not determined, *CTRL* = no enzyme. **f** BFP disruption data using 11 distinct targets (sRGNs guide_5-9 and 11-16, Spy guide_19-23 and 25-30). Data are presented as minimum to maximum values; the box encompasses the 25th to the 75th percentiles, the line in the box is the median. *N* = 6 independent biological replicates for each guide. Significance was determined by two-way ANOVA and Dunnett’s multiple comparison test, ***p < 0.0001, ns = not significant (*p* = 0.8209). Source data are presented in the source data file.

To improve the editing efficiency and specificity of SluCas9, we applied DNA family shuffling. We fragmented and reassembled the above four “parental” genes at multiple areas of high sequence identity, resulting in two combinatorial sRGN DNA libraries with a diversity of 8 × 10^3^ and 1.3 × 10^5^, respectively (Fig. 2a). To retain favorable 5′-NNGG-3′ PAM recognition, the PAM-interacting domain (PID) in all library variants was initially held constant as the native SluCas9-PID. These libraries were prescreened using a “live/dead” bacterial survival assay, yielding an enriched library of 1824 active nucleases, which were further narrowed to 165 hits with superior cleavage kinetics using a cell-free cleavage assay (Fig. 2b, Supplementary Fig. 3a, b). These resulting 165 hits were subsequently assayed for activity in mammalian cells by BFP gene disruption (Fig. 2c). In line with previous observations^25^, we found generally weak correlation between the activities of a given engineered variant in the different screening assays (Fig. 2d). We thus selected the two top hits for each assay, respectively designated sRGN1-4, for further engineering (Supplementary Fig. 4a).

Further improvements in performance were explored by incorporating alternative PIDs. As stated above, we initially replaced PID domains of wild-type ShyCas9 with alternative Slu-PID fragments, resulting in chimeric nucleases that acquired the ability to cleave a 5′-NNGG-3′ PAM-containing target, in contrast to the Shy-native 5′-NNARMM-3′ PAM (Supplementary Fig. 4b). This motivated replacement of respective C-terminal fragments of the selected sRGNs with analogous fragments from either SpaCas9 or SmiCas9, since both also recognize a 5′-NNGG-3′ PAM. While tested SmiCas9 PID fusions were inactive, the SpaCas9 C-terminal fragments resulted in variable activity, depending on the recipient sRGN. All three SpaCas9-PID fragments resulted in active sRGN3 fusions, yielding sRGNs 3.1–3.3, and increased the median BFP-cleavage activity up to 2.8-fold (Fig. 2e). Substituting these PID fragments in the other three top variants yielded no similar benefit (Fig. 2e).

To assess the overall performance with a diverse set of sgRNAs, we then selected sRGN1, sRGN2, sRGN4, and sRGN3.3 for further testing on 11 targets distributed within the *BFP* locus. *BFP* protospacers were selected for either “NNGG” PAM for sRGNs and “NGG” PAM for SpyCas9, resulting in target sites shifted by one nucleotide. While plasmid transformation comparisons are suboptimal for detailed quantitative comparisons, sRGN1, 2, and 4 showed significant activity on most targets. Variant sRGN3.3 demonstrated higher cleavage activity in this assay, comparable to the highly active SpyCas9 (Fig. 2f).

SpyCas9 has been characterized to be, for practical purposes, a single-turnover enzyme, while the smaller SauCas9 exhibits multi-turnover activity^26^. In an in vitro assay with a twofold molar excess of substrate: RNP, sRGN3.1, and its parent, SluCas9, effected near-complete substrate-to-product conversion, indicating multi-turnover capability. For SpyCas9, in contrast, we confirmed single turnover activity (Supplementary Fig. 5a).

With few exceptions, insertion/deletion (indel) pattern analysis of SluCas9, sRGN3.3, and SpyCas9 revealed similarities among these nucleases on the same targets (Supplementary Fig. 5b), supporting previous observations that indel formation pattern is more dependent on target locus than employed nuclease^24^.

### Synthetic RNA-guided nuclease (sRGN) activity and specificity in cell-free and mammalian cell line assays

**Fig. 3:**
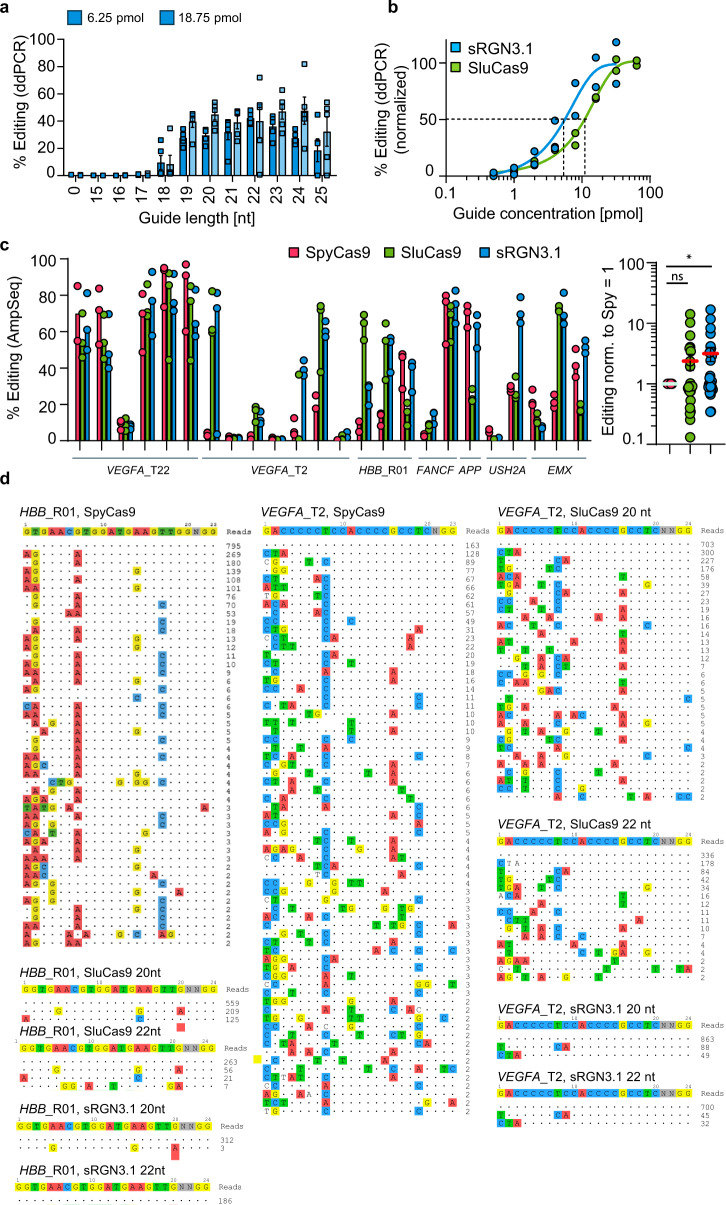
Genome editing efficiency and specificity of SluCas9 and sRGNs at endogenous loci in mammalian cells. **a** The effect of guide length on sRGN3.1 editing efficiency. The *HBB*_R01_T2 site was targeted by sgRNAs of lengths from 15 to 25nts (guide_80-90) using RNP nucleofection and two different RNP concentrations. The editing efficiency was quantified by ddPCR; data are presented as mean ± SEM with *n* = 3 independent biological replicates. **b** Guide concentration dose-response. SluCas9 and sRGN3.1 activity after RNP nucleofection at the *HBB*_R01 locus (guide_87) in HEK293T cells. Curve fitting was conducted by the least-squares methods, r2 = 0.92 (sRGN3.1) and 0.98 (SluCas9), the editing efficiency was measured by ddPCR and normalized to the maximum for each nuclease, data are presented as *n* = 2 independent biological replicates. EC50s were 5.7 pmol for sRGN3.1 and 12.1 pmol for Slu. **c** The median activity on 24 genome targets. Comparison of genome editing efficiency at endogenous targets in HEK293T cells following RNP nucleofection. Twenty-four different guides (5′-NGGG-3′ PAM), across seven genomic loci (*VEGFA, HBB, FANCF*, Apolipoprotein (*APP*), *USH2A,* and *EMX*), were assessed by amplicon sequencing (22 nt guides for SluCas9 and sRGN3.1: guides_32-55; 20 nt guides for Spy: guides_56-79, Supplementary Table 1). SpyCas9, SluCas9, and sRGN3.1 without guide RNA served as controls and had a median editing of 1.08 ± 0.64%; data are presented as *n* = 3 independent biological replicates. The right data from the left panel with average editing on each target normalized to SpyCas9. Red line = mean. The significance was determined using the one-sample Wilcoxon signed-rank test against a theoretical median of one with an alpha of 0.05, ns = not significant, (*) = *p* ≤ 0.05, SpyCa9s vs SluCas9 *p* = 0.68, SpyCas9 vs sRGN3.1 *p* = 0.05. **d** GUIDE-Seq analysis for SpyCas9, SluCas9, and sRGN3.1 on *HBB*_R01 and *VEGFA*_T2. On-target sequences in 5′–3′ orientation are shown in the top row of each panel. Identified off-targets are listed in the subsequent rows and ranked by the number of reads. Matches to the on-target are shown as (.), mismatches are highlighted. Note that for the SpyCas9 *HBB*_R01 off-target analysis, only 47 of the 82 detected off-targets are shown on distinct rows, because 37 of the determined off-target loci are distributed at two identical off-target sequences (AGgaacAtggatgaagCtgg was found 27 times, AGgaacAtggatgGagttgg 10 times). These duplicates were combined into the respective two rows. The full off-target list for each sample can be found in the source data file. Data are presented as *n* = 1 for each guide.

RNPs are attractive, therapeutically relevant entities for ex vivo cellular genome editing, especially due to their limited duration of activity resulting from the faster cellular turnover of Cas9 proteins vs. rAAV genomic DNA or plasmids. Using RNPs, we determined 22 nt as the optimal guide length for sRGN3.1 (Fig. 3a) and detected approximately two-fold higher potency for sRGN3.1 over SluCas9 at the *HBB*_R01 target in mammalian cells in gRNA titration experiments (Fig. 3b). Additionally, higher cleavage activity of sRGN3.1 and sRGN3.3 compared to SpyCas9 was observed on the albumin locus in a murine hepatoma cell line (Supplementary Fig. 6). We next assessed editing on a panel of endogenous, superimposed SpyCas9 and sRGN targets (adjacent to 5′-NNGGG-3′ PAMs) on 24 diverse targets in HEK293T cells (Fig. 3c). The average editing efficiency across these targets was 2.3-fold (SluCas9) and 3.1-fold (sRGN3.1) higher than for SpyCas9 (Fig. 3c, right panel). Additionally, we performed a cell-free FP assessment on 48 synthetic target sequences engineered to have between 20 and 80% GC content, and on 48 additional therapeutically relevant human genomic targets. Overall, we found the protein-engineered sRGN3.1 outperformed SpyCas9 on 76 of these 96 targets and outperformed its most active parental nuclease, SluCas9, on 62 of 96 targets (Supplementary Fig. 7a).

Off-target (OT) cleavage is an important parameter in clinical applications. To initially characterize our sRGNs’ propensities for OT cleavage, we analyzed their cleavage behavior in vitro using each possible single-nucleotide gRNA mismatch along the entire *HBB_*R01 target sequence (Supplementary Fig. 7b). Confirming previous reports^27^, we observed that SpyCas9 showed low mismatch discrimination (low specificity) outside of its PAM-proximal “seed” region. SluCas9 showed overall specificity similar to SpyCas9 but with a less pronounced seed region and higher specificity in the PAM distal region. sRGN3.1 also lacked a prominent seed region, but instead showed an improved average level of mismatch discrimination across the entire protospacer region in comparison to SpyCas9.

To probe the specificity of sRGN3.1, SluCas9, and SpyCas9 in a human cell line, GUIDE-Seq^28^ was employed. We selected well-characterized guides targeting the *VEGFA*_T2^29^ or *HBB*_R01_T2^30^ locus and dosed RNP activity to achieve similar on-target editing for each nuclease (Supplementary Fig. 7d). As expected, the on-target in each case yielded the highest number of reads. For SpyCas9, we recovered 66 unique off-targets using the *VEGFA_*T2 guide (of which 50 had been previously reported^31^) and 82 OTs using the *HBB*_R01 guide. Consistent with our described cell-free observations, we found greatly reduced OT editing for SluCas9 (30 using *VEGFA*_T2, 2 for *HBB_*-R01) and an even greater specificity increase for sRGN3.1 (2 and 1 OTs, respectively). This was even the case in samples for which the extent of on-target editing was greater for sRGN3.1 and SluCas9 than for Spy. Extending the guide length from 20 to 22 nt further reduced the number of OTs from 30 to 15 for SluCas9 on *VEGFA*_T2, and from 1 to 0 for sRGN3.1 on *HBB*_R01_T2 (Supplementary Fig. 7d and Fig. 3d). sRGN3.1 thus displayed significantly higher specificity than SpyCas9 in this context.

### In vivo genome editing by sRGNs using LNP and AAV

Lipid nanoparticles are a current modality of choice to transiently deliver nucleases for genome editing applications in vivo. We thus evaluated the performance of sRGNs when delivered as mRNA in LNPs. Analyses using UPLC (Supplementary Fig. 8a) and cryoTEM (Supplementary Fig. 8b) showed differences in final lipid composition and improved LNP morphology, which appeared to be payload-dependent and which we hypothesize were due to the smaller sRGN mRNA. Interestingly, the sRGN-LNPs also displayed enhanced functional stability, retaining potency significantly longer than SpyCas9-LNPs. Over 9 days of storage at 2–8 °C, a 3.6-fold drop in editing efficiency was observed with SpyCas9-LNPs, whereas a potency drop of only 1.3-fold was observed with sRGN-LNPs, resulting in an over 3.6-fold superiority in in vivo editing efficiency for the sRGN over SpyCas9 after 9 days of storage (Supplementary Fig. 8c).

**Fig. 4:**
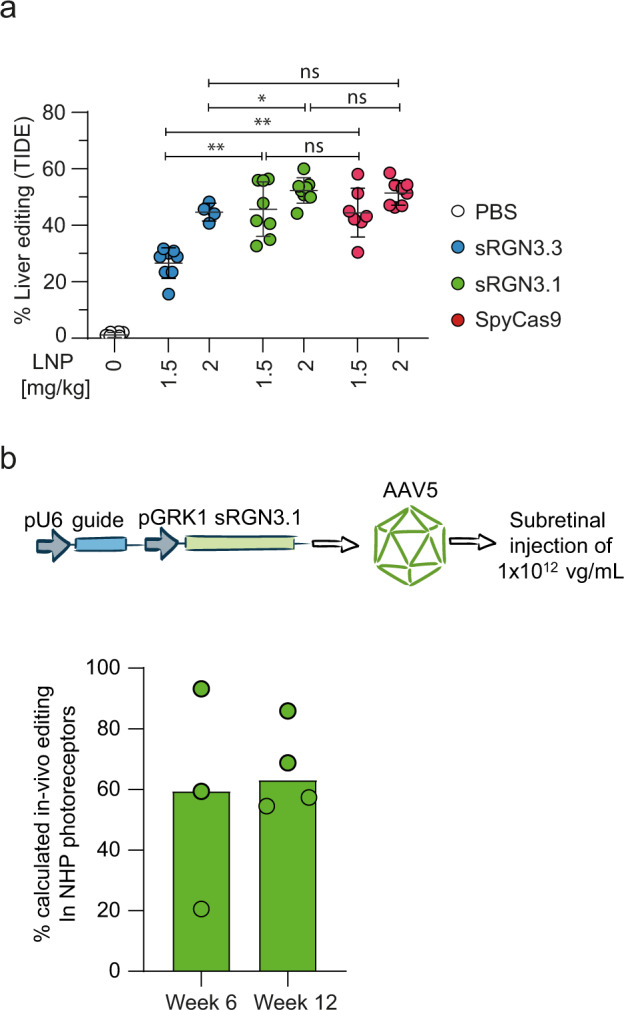
In vivo delivery of sRGNs by LNP or AAV to mouse and nonhuman primate. **a** Liver editing in mouse using sRGN-LNPs and SpyCas9-LNPs at doses of 1.5 and 2 mg/kg. LNPs or PBS control was administered intravenously. Data are presented as mean ± SD, each dot represents an independent biological replicate; sRGN3.3: *n* = 8 at 1.5 mg/kg and *n* = 4 at 2 mg/kg, sRGN3.1: *n* = 8 at both doses, SpyCas9: *n* = 7 at 1.5 mg/kg and *n* = 8 at 2 mg/kg. All mRNA constructs were m1Ψ base-substitution modified. All mRNAs used in this work were of comparable quality: full-length purity ≥85% and low dsRNA levels effected by reverse-phase purification. sRGN3.3 and sRGN3.1 used the same end-modified sgRNA against albumin (*Alb*-T1), while SpyCas9 used a similar sgRNA with a protospacer shifted by one nucleotide (to accommodate “NGG” PAM instead of “NNGG”, sequences in Supplementary Table 1) and internal 2′O-methyl modifications (see Supplementary Fig. 8e). Significance was tested using the Kruskal–Wallis test (alpha = 0.05) corrected for multiple comparisons using Dunn’s test; *p < 0.05; **p < 0.01; ns = not significant (1.5 mg/kg: sRGN3.3 vs 3.1: *p* = 0.0027, sRGN3.1 vs Spy: *p* > 0.9999, sRGN3.3 vs Spy: *p* = 0.0053; 2 mg/kg: sRGN3.3 vs 3.1: *p* = 0.0449, sRGN3.1 vs Spy: *p* > 0.9999, sRGN3.3 vs Spy: *p* = 0.0780). **b** top: schematic of the genetic construct, packaging, and delivery of AAV5 vector. Bottom: in vivo editing of nonhuman primate (NHP) photoreceptors by subretinal injection of rAAV5 vectors. AAV5 vectors carrying sRGN3.1 and WT-IVS40 sgRNA (guide_111) were injected into the subretinal space of NHPs. Indels were quantified by amplicon sequencing from retinal punches. Individual measurements and bar as mean, *n* = 3 eyes at 6 weeks and *n* = 4 eyes at 12 weeks, independent biological replicates. A previous study and our internal assessment demonstrated that nucleases driven by a photoreceptor-specific GRK1 promoter are expressed only by photoreceptors, which account for approximately 30% of the cells in retinal punches^60,61^. We therefore calculated the frequency of indels in the photoreceptor fraction with a multiplier of 3.3. Source data are provided in the source data file.

We next compared LNP-mediated editing of the albumin locus by sRGNs and SpyCas9 in cells, as well as in the liver of C57BL/6 mice (Fig. 4a). Similar to the performance in cells (Supplementary Fig. 6), LNPs harboring sRGN3.1 mRNA displayed editing comparable to SpyCas9 in vivo, while sRGN3.3 was less efficient in this experiment. In addition, we tested the in vivo effects of mRNA sequence and chemistry modifications, including uridine depletion^32^ and base substitution of uridine. Modification with (N1)-methylpseudouridine (m1Ψ) or with pseudouridine (Ψ) had only modest influence on in vivo editing outcome (Supplementary Fig. 8d). Alternative modifications of the sgRNAs enhanced in vivo performance, with the utilization of a 23-mer protospacer showing the largest effect on gene editing efficiency (Supplementary Fig. 8e).

In addition to LNP delivery, we evaluated AAV-based delivery, since the smaller size of sRGNs provides a packaging advantage over the larger SpyCas9. Usher syndrome type II (USH2) is an autosomal recessive disorder leading to hearing loss and retinitis pigmentosa^33^. This is caused by mutations in the *USH2A* gene, which is expressed in cochlear hair and retinal photoreceptor cells. One commonly found mutation in USH2 patients (termed IVS40) results in the insertion of an additional exon in *USH2A* mRNA, leading to premature truncation of the gene product^33^. We used sRGN3.1 and SpyCas9 to address the IVS40-specific mutation in an IVS40 homozygous cell line (referred to as 293FT-IVS40, also see Methods section). On-target editing in the 293FT-IVS40 line was higher for sRGN3.1 than for SpyCas9 (Supplementary Fig. 9b). Upon examining specificity by GUIDE-Seq and targeted amplicon sequencing of OT sites with up to seven nucleotide substitutions from the on-target site, we identified no OT editing above background for either SpyCas9 or sRGN3.1 (see amplicon sequencing data in the source data file, tab Supplementary Fig. 9a).

Toward clinical applications in humans, we tested the editing efficiency of rAAV-delivered sRGN3.1 in nonhuman primate (NHP) photoreceptors (PR) in vivo. As our NHP model possessed only the WT-*USH2A* allele, we pursued a surrogate strategy, assessing sRGN3.1 activity using a WT-specific *USH2A* guide (Supplementary Fig. 9b).

Of note, the larger size of SpyCas9 precludes an all-in-one approach. Comparison with SpyCas9 would require generation and co-injection of two rAAV5-genomes, one encoding the SpyCas9 nuclease and the second encoding the guide RNA expression components. Ethical considerations of a poorly comparable control in the NHP model thus led us to exclude SpyCas9 from this experiment.

The smaller size of sRGN3.1 allowed its packaging together with sgRNA into single AAV5 particles. We injected these particles into the NHP subretinal space and quantified indels in PRs. Six and twelve weeks after injection, we counted robust sRGN3.1-mediated in vivo editing in NHP-PRs (Fig. 4b). In summary, this result establishes successful AAV delivery of the compact sRGN3.1 gene, which was co-packaged with its sgRNA in single AAV vector particles, and allows for substantial genome editing in NHP photoreceptor cells.

## Discussion

Here we report the generation and characterization of programmable small RNA-guided nucleases, which are smaller than SpyCas9, but with hgiher activity and specificity. Mouse studies with LNPs and AAV-mediated gene therapy in NHP show the potential of these additions to the CRISPR toolbox.

The packaging size limitation of AAV vectors and potential benefits from formulating smaller mRNA payloads into LNPs has generated considerable interest in genome-editing nucleases smaller than the widely used SpyCas9^34,35^. However, these smaller alternatives to SpyCas9 typically show lower editing efficiencies on average across diverse targets in human cells and/or possess more complex PAM sequences, which further limit their utility^18,36,37^. SauCas9 is similar in size to the presently described sRGNs, but has a less advantageous PAM, and engineered forms of SauCas9 struggle with lower activity on certain targets^38^. Of note, Kleinstiver et al. observed an average editing of 24.7% across 55 targets sites, while sRGN3.1 presented here shows an average editing of 41% across the 24 tested target sites. CjeCas9, although 74 amino acids smaller^17^, has not reached widespread use, potentially due to its long and degenerate PAM, which reduces the number of genomic sites targetable. Additionally, a recent paper in Science Advances^39^ raised important concerns about the enzyme, as it was shown to exhibit guide-independent, nonspecific host-cell DNA damage. In contrast, Nme2Cas9 is a small “CC” PAM nuclease. Importantly, unlike the sRGNs described in this work, the authors of the Nme2 paper find Nme2 inferior to SpyCas9 on 24 out of 28 sites tested^40^. Other recently published hypercompact enzymes such as Cas14 and CasPhi^41,42^ are of interest in the field, even if no genome-editing activity on native (i.e., endogenous) genes in mammalian cells has yet been reported and no head-to-head comparison with SpyCas9 has been conducted. In addition, time fluorescence disruption activity assays like the ones reported here suggest they may be inferior to SpyCas9. Furthermore, we are not aware of reports outlining the specificity of CasPhi in a mammalian setting. Finally, the presence of a single nuclease domain in this protein makes it unlikely that it can be modified for the strand-specific nickase activity required for base editing or prime editing applications. By contrast, sRGN3.1, the product of this work, constitutes a chimeric dual-nuclease domain enzyme that recognizes a short 5′-NNGG-3′ PAM, and which outperformed SpyCas9 regarding activity and specificity on the majority of tested targets.

We are aware of no reports to date of an alternative Cas9 nuclease with on-target editing efficiency comparable to SpyCas9 across a range of genomic targets. sRGN3.1 was determined to have a median activity 17% higher than SpyCas9 on 24 targets selected for analysis only by virtue of an associated NGGG motif. Using LNPs delivered i.v. to the mouse liver, we demonstrated that the in vivo albumin-locus target activity of sRGNs was comparable to SpyCas9. However, this comparison employed a 20 nt guide for both nucleases, rather than the optimal guide length for sRGN3.1. In separate experiments (Fig. 1b and Supplementary Fig. 8e) median sRGN activity in vivo was boosted significantly with 23 nt guides. In addition, RNA base modifications were also shown to boost sRGN activity in vivo. Together, these results indicate sRGNs are an attractive payload that can be effectively delivered by LNPs for robust in vivo gene editing.

High specificity is of critical importance in gene therapy applications. The undesirable properties of off-target (OT) activity^43,44^ and the resulting undesired mutations and/or chromosome rearrangements^45^ have been attributed to the high activity of SpyCas9. Nevertheless, data on the small SauCas9 are suggestive that this lower-activity nuclease has similar specificity to SpyCas9^16^. Our GUIDE-Seq experiments revealed specificity for sRGN3.1 that was markedly higher than for SpyCas9, despite both being titrated to similar on-target editing efficiency. Indeed, these observations were made in immortalized cell line backgrounds that have been shown to more sensitively reveal genomic OT propensities of editing nucleases than primary human cells^46–48^. Interestingly, it appears that in engineering higher activity into sRGN3.1, we relaxed stringency at certain PAM-proximal positions in comparison to SluCas9, the highest activity WT nuclease in our parental set, while simultaneously increasing PAM-distal specificity, at least for the target investigated in depth (Supplementary Fig. 7b). While sRGNs and SpyCas9 both performed well in discriminating the few genomic off-targets possessing one or two mismatches to the on-target gRNA (Supplementary Fig. 7b), there will typically be exponentially more genomic off-targets with three or four mismatches than with one or two. Despite the very high-specificity seed region of SpyCas9, the dispersed base-pairing requirement across the sRGN3.1 gRNA appeared to better serve it in discriminating off-targets with multiple mismatches (Fig. 7b, d). Although higher overall specificity was observed for sRGN3.1 in vitro, an additional hypothesis for its much higher specificity in mammalian cells may paradoxically include the engineered sRGN’s higher activity: the requirement for fewer nuclease molecules per cell may contribute to the more rapid elimination of the nuclease and thus less opportunity for off-target effects.

Effective in vivo delivery of genome editing agents remains challenging for some applications, despite recent commercial successes^34,49,50^. We thus investigated LNP and rAAV in vivo delivery approaches for sRGN3.1. sRGN LNPs had comparable or improved analytical and stability profiles to SpyCas9-LNPs. Further investigation is required to understand if the improved properties observed can be attributed solely to the packaging advantages of smaller RNA payloads. The toxicity of LNP formulations has been shown highly dependent on administered lipid quantities^51^, which also may be improved with equal editing activity in smaller RNA payloads (higher RNA stoichiometry per unit LNP mass). In addition, RNA quality in such formulations is also key and mRNA cost and product quality are typically improved with shorter constructs^52^. The smaller sRGNs are thus also more attractive than SpyCas9 from a biopharmaceutical manufacturing and quality perspective.

AAVs present another clear case for the size-based advantages of sRGN payloads. The large size of SpyCas9 requires the packaging of Cas9 and its sgRNA into two separate AAV vectors, increasing manufacturing complexity and potentially decreasing therapeutic efficacy in some use cases^53^. We demonstrated that subretinal injection of sRGN3.1 as an all-in-one rAAV5 affected robust editing in NHP photoreceptors in vivo.

In summary, we engineered potent, small, genome-editing nucleases that recognize a favorable PAM. We demonstrated targeted genome cleavage efficiency equal or superior to the “activity gold-standard” SpyCas9, across a broad selection of targets in human cell lines. In addition, sRGNs showed markedly higher specificity in GUIDE-Seq experiments in human cell lines. Robust in vivo editing efficiency was observed for LNP delivery in mice and AAV5 delivery in NHP. For a given therapeutically relevant genomic locus, our data indicate that sRGN3.1 has a higher probability of being more active and more specific, while offering the delivery advantages of a smaller nuclease. We thus expect that these synthetic RNA-guided nucleases will be valuable additions to the current repertoire of CRISPR gene therapy nucleases, and look forward to the implementation of these enzymes in a range of applications.

## Methods

### Identification of Cas9 sequences

Four uncharacterized, putative Cas9 nucleases with ~80% amino acid similarity and ~65% amino acid identity from shotgun sequencing data in Uniprot for four *Staphylococcus* species were identified: *S. hyicus* (Shy) GB: CP008747.1, Uniprot: A0A418JLD8, *S. lugdunensis* (Slu) NCBI reference sequence: NZ_GL622352.1, Uniprot: A0A133QCR3, *S. microti* (Smi) GB: JXWY01000132.1, Uniprot: A0A0D6XNZ8, and *S. pasteuri* (Spa) GB: CP004014.1 (see also Supplementary Table 2). Direct repeat and tracr sequences were identified by inspection. We exploited GAAA tetraloop fusion of crRNA and tracrRNA, as previously reported^5,16^, to form single-guide RNAs (sgRNAs). DNA sequences for sgRNAs and nucleases were codon optimized and ordered at GeneArt and Twist Biosciences.

### Screening of libraries

In an initial “live/dead” (L/D) cell survival selection in *E. coli*^54^, we depleted nonfunctional or low-activity variants (Supplementary Fig. 3b and 3c). E.coli BW25141(λDE3) cells harboring a plasmid-based arabinose-inducible ccdB reporter gene were co-transformed with an expression plasmid for sgRNA (targeting VEGFA-T2 within the ccdB reporter) and an IPTG-inducible nuclease expression plasmid. Cells were recovered for 60 min in SOB media and subsequently plated on LB selection plates (chloramphenicol and 10 mM arabinose). Functional Cas9 variants cleaved the toxic ccdB reporter gene and led to cell survival under selection conditions. In all, 1824 functional clones from the L/D selection were expressed in *E. coli* and lysates were individually tested for activity in a biochemical oligodeoxynucleotide (ODN) cleavage assay (Supplementary Fig. 3d).

### E. coli-based negative selection assay for identification of Cas9 PAM specificities

The negative selection for identification of PAM specificities of generated Cas9s was done as previously described^54^. Briefly, competent *E. coli* BL21Star(λDE3) containing an IPTG inducible Cas9 (Amp^R^) and a sgRNA (Kan^R^) expression plasmid were electroporated with a library of plasmids (Cm^R^) harboring a *VEGFA_*T2 target sequence with 3′ adjacent N_7_ PAM sequences. Plasmids containing functional PAMs are depleted when Cas9 is induced, whereas plasmids harboring nonfunctional PAMs remain uncleaved. Following a 60 min recovery in SOB media, transformations were plated on LB plates in two sets: one induced (ampicillin, kanamycin, chloramphenicol, and 0.2 mM IPTG) and one not induced (ampicillin, kanamycin, chloramphenicol) set. In order to exceed the complexity (16,384) of the N_7_ library, approximately 100,000 CFUs were plated, and plasmids were isolated using Plasmid DNA Midiprep Kit (Qiagen). From the resulting plasmid libraries, a 271 bp fragment covering the *VEGFA_*T2 target and N_7_ PAM was amplified by PCR using KOD Hot Start DNA Polymerase (Merck Millipore) with primers fw: 5′-CTCAGAAGTGAAACGCCGTAGCG-3′ and rev: 5′-CTTTTGAGTGAGCTGACACCGCTC-3′ followed by a PCR-product purification step with QIAquick PCR Purification Kit (Qiagen). Single-indexed libraries were prepared using the TruSeq DNA PCR-Free High Throughput Library Prep Kit (Illumina). Next-generation sequencing (NGS) was performed using a MiSeq reagent kit v3 with 600 bp paired-end sequencing on an Illumina MiSeq Sequencer. The resulting raw FASTQ files from the MiSeq runs were analyzed with a python script to determine relative PAM depletion. Shortly, this involves scripts to (1) process crude NGS Data for *VEGFA_*T2 target sequence [Each FASTQ entry is scanned for 12 constant nucleotides on the library amplicon on both strands. If the constant region is found, then the seven variable nucleotides flanking the protospacer region are captured and multiples are counted.]; (2) calculate depletion values by comparing PAM sequence motifs of depleted samples to undepleted controls and; (3) rank sequences according to frequency.

### Fluorescence polarization assay

Oligonucleotide duplexes (oligo sequences are listed in Supplementary Table 3) were prepared in 1× PBS + 5 mM MgCl_2_ as 10 µM solutions (from 100 µM stocks) by melting at 95 °C for 5 min and then slowlycooling to room temperature (RT). The stocks were subsequently diluted in 1× PBS + 5 mM MgCl_2_ + 0.05% pluronic F68 (Sigma Aldrich) to 20 nM working solutions. Twenty microlitres (20 nM) of dsDNA was immobilized on a streptavidin-coated plate (Greiner, 384-well) and incubated for 10 min at RT. The plate was washed twice with 1X PBS and was subsequently incubated with 20 µL of a diluted lysate supplemented with 60 nM sgRNA targeting the *VEGFA_*T2 target sequence. Cas9 variants were expressed in 800 µL TB (Terrific Broth)-medium, as described in the protein expression and purification section, at the 96-well expression scale. Cells were harvested and lysed in 120 µL lysis buffer (1× PBS + 0.5 × BugBuster® + 5 mM MgCl_2_) and incubated for 10 min at RT under shaking conditions. The lysate was cleared by centrifugation: 3166 × *g*, 10 min at 4 °C. Cleared lysate was diluted 1:3.5 (v/v) in 1× PBS + 5 mM MgCl_2_ and mixed with 60 nM sgRNA, prior to a 5 min incubation at 37 °C. Cleavage was monitored by following both decreasing anisotropy and increasing fluorescence intensity for 60 min (excitation wavelength: 635 nm; emission wavelength: 670 nm) at 37 °C in a plate reader (Tecan Infinite M1000 pro). For activity specificity assessment of purified protein, prior to the kinetic measurement, RNPs were formed in 1× PBS + 5 mM MgCl_2_, with a two-fold excess of sgRNA at 37 °C for 5 min with adjusted protein levels for the same activity. To obtain an overall specificity value, the cleavage kinetics were analyzed by calculating the initial slope of the reaction. The slopes were calculated for each of the 61 substrates (including the un-modified sequence and all single-nucleotide exchanges along the *VEGFA_*T2 target sequence) and normalized to the value of the on-target substrate. For position-specific nucleotide tolerance profiles, the cleavage kinetics were analyzed by calculating the initial slope of the oligonucleotide cleavage reaction. The slopes were calculated for each of the 61 substrates and normalized to the value of the on-target substrate (defined as 1). The normalized cleavage values for all 60 off-target substrates were grouped according to their position. For each of the 20 nucleotide positions in the target sequence, the normalized cleavage value of the three single nucleotide mismatches was plotted to illustrate the position-specific nucleotide tolerance of the Cas9 protein.

### Expression and purification of Cas9 proteins

Cas9 proteins were expressed from a plasmid harboring a TRC-promoter expression cassette, encoding an N-terminal 6xHis-MBP-TEV fusion followed by a nucleoplasmin NLS and a C-terminal SV40 NLS. *E. coli* BL21 (DE2) star was transformed with the expression construct and a single colony was grown overnight at 37 °C in ZY-medium (including 21.5 ml of 52 × 5052 solution (0.5% glycerol, 0.05% glucose, and 0.2% α-lactose), 52.5 ml of 20xNPS, and 2 mM MgSO_4_) supplemented with 100 µg/mL ampicillin. One liter of supplemented ZY-medium was inoculated 1:100 (v/v) with the overnight culture and shaken at 37 °C for 3 h. After 3 h, the culture was cooled to 18 °C and incubated overnight. IPTG in a final concentration of 1 mM was added, and the culture was incubated for two additional days. The cells were harvested by centrifugation (4376 × *g*, 20 min, 4 °C), the cell pellet was resuspended in 22 mL wash buffer (50 mM Na_2_HPO_4_ 300 mM NaCl, 10 mM imidazole, pH 8.0) and lysed under sonification 10 min on ice (amplitude 30). The lysate was cleared by centrifugation (194300 × *g*, 30 min, 4 °C) and applied to His-Trap HP columns (GE healthcare) using the ÄKTA pure system (GE healthcare) to perform an immobilized metal affinity chromatography (IMAC) step. After two washing steps, first with wash buffer including 0.1% triton, then with wash buffer only, the protein was eluted with a linear gradient of 10–500 mM imidazole. Pooled protein fractions were subsequently treated with TEV at 4 °C for 2 days to cleave off the His-MBP tag. After 48 h, the protein solution was buffer exchanged into equilibration buffer (20 mM HEPES, 100 mM KCl, pH 7.0) using PD-10 columns (GE healthcare) and a cation exchange chromatography was performed by using HiTrap SP HP columns (GE healthcare) with a linear gradient of 100–1000 mM KCl (20 mM HEPES, 1000 mM KCl, pH 7.0). Before protein fractions were pooled, quality and purity were checked via SDS-PAGE, and a biochemical cleavage assay was conducted to exclude fractions with inactive nuclease contaminations. Nuclease activity used in downstream assays was standardized by sgRNA titrations. Briefly, starting from an excess of sgRNA, the amount of sgRNA was reduced, until a decrease in activity was observed. Fractions were pooled, concentrated, and stored in storage buffer (20 mM HEPES, 200 mM KCl, 1 mM DTT, 40% glycerol pH 7.0) at –20 °C.

### Multiple-turnover analysis

RNP formation was performed with 20 µL of gRNA (800 nM stock) in 20 µL of 1x NEB buffer and heated to 90 °C for 3 min and cooled to 25 °C at 1 C/s. RNP was formed by combining 20 µL of this gRNA with 20 µL of Cas9 in 1x NEB buffer at a stock concentration of 200 nM the final concentration was 100 nM Cas and 200 nM gRNA. The concentration of active Cas9 was determined^55^ before kinetic analysis for enzymes that were obtained commercially or synthesized in house. This solution was incubated at 25 °C in a thermal cycler for 30 min prior to use. *On-target reactions*: 8 µL of off-target plasmid (250 nM stock) was combined with 10 µL of 10x NEB buffer and 72 µL of RNase-free water. This solution was placed on a heat block at 37 °C and the reaction was initiated by adding 10 µL of the RNP stock. *Quenching and analysis*: At various time points, 5 µL of the reaction was removed and quenched with 2 µL of a 500 mM EDTA solution. Once all samples were collected, RNP was removed by adding 1 µL of proteinase K to each reaction. Samples were analyzed on an Agilent 2100 Bioanalyzer using a DNA 7500 kit.

### Cell culture

HEK293T cells (ref. CRL-3216) and murine Hepa 1–6 cells (ref. CRL-1830) were purchased from ATCC and 293FT cells were purchased from Thermo Fisher Scientific (ref. R70007).

HEK293T, 293FT, and homozygous 293FT-IVS40 cell lines were cultured in DMEM-GlutaMAX (Gibco) supplemented with 10% FBS (Gibco), and 1% sodium pyruvate (Gibco) and were cultured at 37 °C in 5% CO_2_. Hepa 1–6 cells were cultured in DMEM (Gibco) supplemented with 10% FBS (Gibco) and 1% penicillin-streptomycin (Gibco) at 37 °C with 5% CO_2_.

293FT-IVS40 cell lines were constructed as follows: to introduce the single nucleotide substitution corresponding to IVS40, TrueCut Cas9 Protein v2 complexed with TrueGuide synthetic sgRNA (Thermo Fisher Scientific) and 200-nucleotide ssODN (5′-TCAGCCAGAGCAGGAAGCTAATAAAATGTATGCTGGCTTTTAAGGGGGAAACAAATCATGAAATTGAAATTGAACACCTCTCCTTTCCCAAGGTAAGAGATCATCTTTAAGAAAAGGCTGTGTATTGTGGGGGTTTGAAGTGCAAGTTCATCTCATTATCATGGATGTTTCACCCATAATACTATCATCATATGCAGGAG -3′) were used for stable transfections. Transfected cells were cultured as serial dilutions and screened by Sanger sequencing to isolate homozygous clones.

### Plasmid, mRNA, and RNP transfection, harvesting, and lysis

For BFP disruption assay and indel pattern analysis, 15,000 HEK 293T cells/well were seeded the day before transfection in poly-d-lysine-coated 96-well plates (Biocoat Poly-d-Lysine, Fisher Scientific). On the next day, a medium change was performed, which was followed by the transfection of 140 ng Cas9- or sRGN-expression plasmid plus 60 ng gRNA-expression plasmid using LipoD293 (SignaGen) as a transfection reagent according to the manufacturer’s instructions. The medium was changed 24 h later. Seventy-two hours post transfection medium was removed, and cells were trypsinized in 25 µL TrypLE (Gibco) for 15 min at 37 °C and diluted with 100 µL PBS. Ten microliters of the sample was lysed by adding 0.5 µl DNA Release additive (B93, Thermo Fisher Scientific) and 20 µL Dilution buffer (F1325ML, Thermo Fisher Scientific) and incubated for 5 min at room temperature followed by 98 °C for 2 min. For mRNA transfection, murine Hepa 1–6 cells were plated in 24-well plates at a density of 50,000 cells per well. After incubation for 2 hr, cells were transfected with 0.25 µg of nuclease mRNA and 0.25 µg of sgRNA per well using Lipofectamine MessengerMAX (Thermo Fisher Scientific) by following the manufacture′r’s instructions. Cells were collected 3 days after transfection and genomic DNA was extracted using the DNeasy Blood & Tissue Kit (Qiagen) following the manufacturer’s instructions. Parental 293FT and the homozygous IVS40 293FT cell line (2 × 10^5^ cells) were transfected with 2 µg of a sRGN3.1 expression plasmid and with either T428 sgRNA or T428 surrogate sgRNA by using Lipofectamine 3000 (Thermo Fisher Scientific Scientific). Genomic DNA was extracted 7 days after transfection with DNeasy Blood & Tissue Kit (Qiagen). A fragment containing the editing region was amplified using PCR with the appropriate primers (Supplementary Table 3) and used for TIDE analysis.

For Ribonucleoprotein (RNP) complex formation, crRNA and tracrRNA (IDT, phosphorothioate bonds, and 2'-O-methyl groups at the last two nucleotides, see Supplementary Table 1 for sequences) were dissolved in IDTE buffer at 200 µM and mixed at equimolar ratios to obtain 100 µM working solution. The crRNA:tracrRNA duplex was incubated at 95 °C for 5 min and gradually cooled to 4 °C. To prepare Cas9/sRGN-RNP complexes, Cas9/sRGN protein was incubated with crRNA:tracrRNA duplex at a 1:2 molar ratio. Cas9/sRGN and RNA complex was incubated in 10 mM TRIS at RT for 15 min to form RNP complexes. 2 × 10^5^ cells were washed with 1X PBS (Gibco), detached with Accutase (Sigma), spun down by centrifugation at 300x*g* for 5 min, washed again with 1X PBS and nucleofected with precomplexed RNPs using the 4D nucleofector with the SF cell line kit and program CA-137 (Lonza), according to the manufacturer’s protocol. Cells were seeded on poly-d-lysine-coated 24-well plates (Biocoat Poly-d-Lysine, Corning) and harvested 2–3 days after nucleofection by 15 min TrypLE (Gibco) incubation at 37 °C. Genomic DNA from RNP-treated cells was extracted with QIAamp DNeasy Blood & Tissue Kit (Qiagen) according to the manufacturer’s instructions.

### BFP disruption assay

This assay was adapted based on the previously described traffic light assay^56^. Briefly, HEK293T cells harboring a gene encoding BFP in the *AAVS1* locus were transfected with plasmid expressing Cas9 and a sgRNA targeting the reverse strand of BFP genomic sequence (guide_5 for initial screens). Transfection of plasmids was performed using Lipofectamine 3000 (Thermo Fisher Scientific, Cat no. L3000015) as described by the supplier. Flow cytometric analyzes of BFP signal disruption was carried out 7 days posttransfection as follows. Cells were prepared by washing with 1X PBS, trypsinization, and resuspension in 200 µL FACS buffer (1X PBS supplemented with 2% FCS). BFP signal from minimum of 10,000 cells was assayed using the V450 filter set in the BD FACS Canto II (see Supplementary Fig. 1 for gating strategy). For BFP landscaping analysis sRGNs guide_5-17 and Spy guide_19-31 were used. We switched from the initial T7 promoter spacer (5′-TATA-3′) to the more canonical U6 spacer (5′-AAACACC-3′) for these experiments. Gating strategy is presented in Supplementary Fig. 10.

#### Amplicon sequencing (AmpSeq) for on- and off-target analysis and NGS analysis

To assess the editing efficiency or to evaluate predicted off-targets, a ~200–240 bp fragment flanking the genomic target site was amplified by PCR using Q5 High-Fidelity polymerase (2XMM Master Mix (NEB), 1 µL crude cell lysate or 50 ng purified genomic DNA, and barcoded primers (see Supplementary Table 3 for locus-specific primers). The cycling program was performed according to the manufacturer’s instructions with an annealing temperature of 66 °C and an extension time of 15 s. All PCR reactions were pooled into a library and purified with magnetic beads (AMPure XP beads, Beckman Coulter). Bead purification was performed by applying the following reaction steps. Two micrograms of the pooled amplicons were end-repaired and dA-tailed (NEBNext Ultra II End Repair/dA-tailing Module, NEB) and used for ligation of Illumina indices (Blunt/TA Ligase master Mix, NEB). The concentration was measured using the dsDNA HS Kit (Thermo Fisher Scientific) on a Qubit spectrophotometer and diluted to 4 nM. The library was denatured with 0.2 M NaOH for 5 min at RT and diluted to 10 pM using the buffer provided in the MiSeq 300PE v2 Kit. The sample was sequenced with 12.5 pmol PhiX on a MiSeq Instrument (Illumina). For de-multiplexing an in-house python script was used and individual samples were analyzed using CRISPResso V1.0.13^57^ to extract % NHEJ. A threshold of >20,000 reads was chosen.

#### Evaluation of off-target sites at the *USH2A* locus

Forty-nine sites containing up to five nucleotide substitutions from the T428 target sites were selected for analysis. The homozygous IVS40 293FT cell line (2 × 10^5^ cells) was transfected with 2 µg of a plasmid carrying sRGN3.1 and T428 sgRNA in Lonza SF buffer by nucleofection under Program CM-130, and genomic DNA was extracted 7 days after nucleofection. All the selected sites except one on-target site were successfully PCR-amplified, and indels were analyzed by NGS as described in the section Amplicon sequencing (AmpSeq) and NGS analysis.

### Digital droplet PCR

In order to determine editing rates, ddPCR (Bio-Rad) was performed with 50 ng genomic DNA by using ddPCR supermix for probes (no dUTP, Biorad). Droplet formation was accomplished by (Biorad) QX200 Droplet Generator Amplification was done by using the following primers, probes, and annealing temperatures in a 40 cycle program with an additional extension step of 72 °C (*HBB*_R01: amplification primer fw 5′-catggtgcatctgactcctg-3′, rev 5′-ggtagaccaccagcagccta-3′, NHEJ-sensitive-probe 5′-TGAAGTTGGTGGTGAGGCCCT-3′, reference probe 5′-AGGAGAAGTCTGCCGTTACTGCCCT-3′, annealing temperature 58.5 °C). Analysis was performed on the QX200 ddPCR Reader (Biorad).

### GUIDE-seq analysis

GUIDE-seq experiments were performed as previously described^28^. For off-target analyses on the *USH2A* (IVS40) locus, the homozygous IVS40 293FT cell line (2 × 10^5^ cells) was nucleofected with 5 pmol of dsODN and 2 µg of a plasmid carrying sRGN3.1 and T428 sgRNA in Lonza SF buffer by nucleofection under Program CM-130. Genomic DNA was extracted 3 days after nucleofection using the PureLink Genomic DNA Mini Kit (Thermo Fisher Scientific Scientific), and processed for sequencing as previously described^28^.

Briefly, HEK293T cells were transfected with nuclease and sgRNA as RNPs as described above with addition of 5 pmol phosphorothioate (PS) end-protected double-stranded oligonucleotide (dsODN). For *VEGFA*_T2 and *HBB*_R01 off-target analysis we observed higher activities of SluCas9 and sRGN3.1 compared to SpyCas9 on these targets. Therefore, we used 60 pmol SpyCas9 and 30 pmol each of SluCas9 and sRGN3.1 for targeting *HBB_*R01, except for sRGN3.1 with 22 nt guide, 8 pmol were used. For targeting *VEGFA*_T2, 60 pmol SpyCas9, 8, and 18 pmol sRGN3.1 (for 20 nt and 22 nt guide, respectively) and 6 and 10 pmol SluCas9 (for 20 nt and 22 nt guide, respectively) were used to obtain a similar extent of on-target editing for each nuclease (Supplementary Fig. 7c). Genomic DNA was extracted 48 h upon nucleofection with DNeasy Blood & Tissue Kit (Qiagen) and on-target activity of nucleases was evaluated via amplicon sequencing as described above. Genomic DNA was quantified by Qubit dsDNA BR assay (Invitrogen) and 400 ng sample was sheared with NEBNext UltraII FS enzyme mix (NEBNext UltraII FS DNA Library Prep Kit, NEB) for 10 min at 37 °C, followed by 30 min inactivation at 65 °C. Sample libraries were constructed with NEBNext Ultra II DNA Library Prep Kit (NEB) with adapters and primers previously described^28^. Sample libraries were sequenced with Illumina MiSeq. Similar read-depth samples were analyzed with the open-source software package guideseq^28^ (https://github.com/aryeelab/guideseq commit version c608522), using human genome assembly GRC37/hg19 as reference.

### TIDE analysis

Evaluation of editing efficiencies on the *USH2A* and *Alb* locus was done via TIDE (Tracking of indels by decomposition)^58^ analysis. Targeted-specific amplicon products were generated with 50–100 ng template DNA and primers spanning the target site (listed in Supplementary Table 3). Indels were identified via decomposition of quantitative trace data with the TIDE software (https://tide.nki.nl), using default parameters.

### LNP formulation and characterization

The LNPs used in this study comprised a lipid mixture consisting of C12-200 (amino lipid, Axolabs), 1,2-dioleoyl-sn-glycero-3-phosphoethanolamine (DOPE), DMPE-mPEG2000 (PEG-lipid), and cholesterol at a 52.2:15.6:8.7:23.6 mass ratio, respectively (DOPE, PEG-lipid, and cholesterol from Avanti Polar Lipids). LNPs were prepared by rapid microfluidic mixing (Precision NanoSystems) of mRNA and sgRNA in acetate buffer at pH 4.0 with the lipid mixture suspended in ethanol (3:1 aqueous to organic volume ratio). After mixing, LNPs were diluted and dialyzed into PBS, concentrated as needed using 100k MWCO spin cartridges (Amicon), and 0.2 µm sterile filtered. mRNA production and sequence optimization was performed as previously described^32,59^. gRNA was procured from Synthego (sRGN *Alb*-T1), Avecia (SpyCas9 *Alb*-T1), and Agilent (modified sRGN *Alb*-T1 constructs). LNPs were characterized by dynamic light scattering (DLS) using a Wyatt Nanostar, Ribogreen (Thermo Fisher Scientific), for endotoxin (Endosafe, Charles River), by UPLC (Thermo Fisher Scientific), and cryoTEM (MIT) assays.

### Animals

This study complied with all applicable sections of the Final Rules of the Animal Welfare Act regulations (Code of Federal Regulations, Title 9), the Public Health Service Policy on Humane Care and Use of Laboratory Animals from the Office of Laboratory Animal Welfare, and the Guide for the Care and Use of Laboratory Animals from the National Research Council. The protocol and any amendments or procedures involving the care or use of animals in this study was reviewed and approved by the Testing Facility Institutional Animal Care and Use Committee before the initiation of such procedures. Testing Facility Institutional Animal Care and Use Committee: Charles River Laboratories, Mattawan, Michigan 49071 (Macaca fascicularis) and Mispro Biotech Services, Inc. (C57BL/6j). C57BL/6j mice, age: 6 to 8 weeks old, sex: male, source: bred in captivity at Jackson Laboratories, Bar Harbor, Maine, Origin: Jackson Laboratories, Bar Harbor, Maine. Macaca fascicularis, age: 2–4 years old, weight: 2.5–5.0 kg, sex: male, source: bred in captivity at World Wide Primates, Inc., Origin: Mainland Asia.

### Mouse studies to assess liver editing by LNPs

Animals were housed on a 12:12 h light/dark cycle at an ambient mean temperature of 23 °C and humidity at 50%. Experimental procedures were approved by the Institutional Animal Care and Use Committee (IACUC) review board. C57BL/6 animals (6–8 week-old male mice) were obtained from Jackson Laboratories (Bar Harbor, ME). For all mouse studies, animals were injected with a single intravenous dose of LNP-formulated mRNA and sgRNA through the tail vein, and after 96 h ± 5% mice were euthanized for genome-editing analysis. Whole liver was flash-frozen in liquid nitrogen, homogenized and genomic DNA was extracted using the DNeasy Blood & Tissue Kit (Qiagen) according to the manufacturer’s instructions. A fragment containing the editing region was amplified using PCR and used for TIDE analysis.

### Subretinal injection of AAV5 vectors and analysis of genome editing in photoreceptors of nonhuman primates

The Photoreceptor-specific GRK1 promoter was chosen to limit Cas9 expression to photoreceptors^60,61^. A U6 promoter-driven T428 surrogate sgRNA, and the sRGN3.1 gene under GRK1 promoter were inserted between AAV2 ITRs and packaged in AAV5 capsids. The AAV5 vector was diluted to 1 × 10^12^ or 2 × 10^12^ vg/mL with phosphate-buffered saline containing 0.001% F68. The animals were anesthetized, and the eyes prepared for insertion of two 25-gauge scleral ports 3 mm posterior to the limbus. An endoilluminator probe was inserted in one port and a subretinal injection cannula was inserted through the second port. The subretinal injection cannula was advanced to mid-vitreous and the small diameter injection cannula was advanced until it contacted the retinal surface. AAV5 vector solution of volume 0.1 mL was slowly delivered to induce a subretinal bleb. Once the dose was delivered, the injection cannula and endoilluminator were removed, the scleral ports were removed, and the sclerotomies were sealed using electrocautery.

### Retinal punch biopsy

Following enucleation, the anterior chamber, lens, and vitreous humor from each eye were removed. Four radial cuts were made in the eyecup to flatten the globe. The subretinal bleb and a portion of the neurosensory retina distal to the bleb were collected with a 6–8 mm biopsy punch. The samples were snap-frozen on dry ice. Frozen retinal samples were pulverized using a Geno/Grinder 2010 (SPEX SamplePrep, LLC) at 1500 revolutions per minute for 2 min. The homogenized tissue was suspended in phosphate-buffered saline (PBS) and split into three tubes. Genomic DNA was extracted from approximately one-third of the retinal samples using QIAamp DNA Mini Kit (Qiagen), and indels were quantified by NGS as described above.

Statistical analysis and curve-plotting were conducted using GraphPad Prism 8. Where applicable, normal distribution was determined before suitable downstream statistical analysis using D’Agostino & Pearson test. Unless stated otherwise, *p < 0.05, **p < 0.01, ***p < 0.001.

### Patents

WO2019183150 covers sRGN3.1, SpaCas9, ShyCas9, and SmiCas9 sequences. Status: pending. Applicant: CRISPR Therapeutics AG, Bayer Healthcare LLC. Inventors: COHNEN, André, SCHMIDT, Moritz J., COCO, Wayne M., GAMALINDA, Michael B., GUPTA, Ashish, PITZLER, Christian, RICHTER, Florian, TEBBE, Jan, CHENG, Christopher J., TAKEUCHI, Ryo, REISS, Caroline W.

WO2019118935 covers SluCas9. Status: pending. Applicant: CRISPR Therapeutics AG, Bayer Healthcare LLC. Inventors: COHNEN, André, SCHMIDT, Moritz J., COCO, Wayne M., GUPTA, Ashish, TEBBE, Jan, SCHULENBURG, Cindy, PITZLER, Christian, GAMALINDA, Michael B. JACH, Sabine, RICHTER, Florian, ARUMUGHAN, Anup, SAALWÄCHTER, Corinna.

### Reporting Summary

Further information on research design is available in the Nature Research Reporting Summary linked to this article.

## Supplementary information

Supplementary Information

Supplementary Data 1

Supplementary Data 2

Supplementary Data 3

Supplementary Data 4

Supplementary Software

Reporting Summary

Description of Additional Supplementary Files

Peer Review File

## Data Availability

All data generated or analyzed during this study are included in this published article and its supplementary information files and are available from the corresponding author upon reasonable request. Parental *Staphylococcus* can be accessed on Uniprot. *S. hyicus* (Shy) GB: CP008747.1, Uniprot: A0A418JLD8, *S. lugdunensis* (Slu) NCBI reference sequence: NZ_GL622352.1, Uniprot: A0A133QCR3, *S. microti* (Smi) GB: JXWY01000132.1, Uniprot: A0A0D6XNZ8 and *S. pasteuri* (Spa) GB: CP004014.1. Sequences of engineered proteins used herein are available in Supplementary Table 2. NGS data of this study have been deposited in the Sequence Read Archive (SRA) at NCBI with the project number PRJNA731307. Source data are provided with this paper.

## Source data

Source Data

## Code availability

All published algorithms used in this study are referenced in the Methods section. Custom-made algorithms are available from the corresponding author upon request. Supplementary Software is available with this manuscript.
