# Addendum to: Improved CRISPR genome editing using small highly active and specific engineered RNA-guided nucleases

**DOI:** 10.1038/s41467-023-39141-w

**Published:** 2023-06-13

**Authors:** Moritz J. Schmidt, Ashish Gupta, Christien Bednarski, Stefanie Gehrig-Giannini, Florian Richter, Christian Pitzler, Michael Gamalinda, Christina Galonska, Ryo Takeuchi, Kui Wang, Caroline Reiss, Kerstin Dehne, Michael J. Lukason, Akiko Noma, Cindy Park-Windhol, Mariacarmela Allocca, Albena Kantardzhieva, Shailendra Sane, Karolina Kosakowska, Brian Cafferty, Jan Tebbe, Sarah J. Spencer, Scott Munzer, Christopher J. Cheng, Abraham Scaria, Andrew M. Scharenberg, André Cohnen, Wayne M. Coco

**Affiliations:** 1 Bayer AG, Leverkusen, Germany; 2 Casebia Therapeutics LLC, Cambridge, MA USA; 3 CRISPR Therapeutics INC, Cambridge, MA USA

**Keywords:** Gene therapy, CRISPR-Cas9 genome editing

Addendum to: *Nature Communications* 10.1038/s41467-021-24454-5, published online 9 July 2021

During the peer review of this Article, a related work reporting the discovery and engineering of small SlugCas9 nucleases was published in *Nucleic Acids Research* ‘Discovery and engineering of small SlugCas9 with broad targeting range and high specificity and activity’.

Hu Z. et al. Discovery and engineering of small SlugCas9 with broad targeting range and high specificity and activity, *Nucleic Acids Research*, Volume 49, Issue 7, 19 April 2021, Pages 4008–4019.

