# Supplementary material for: Improved CRISPR genome editing using small highly active and specific engineered RNA-guided nucleases
Source: Nat Commun. 2021 Jul 9;12:4219. doi: 10.1038/s41467-021-24454-5 (PMC8271026; doi:10.1038/s41467-021-24454-5)
Supplement: Supplementary file 1 — Supplementary Information [file 41467_2021_24454_MOESM1_ESM.pdf]

Improved CRISPR genome editing using small highly active and specific engineered RNA-guided nucleases

Moritz J. Schmidt[1], Ashish Gupta[1], Christien Bednarski[1], Stefanie Gehrig-Giannini[1], Florian Richter[1], Christian Pitzler[1], Michael Gamalinda[1], Christina Galonska[1], Ryo Takeuchi[2], Kui Wang[2], Caroline Reiss[2], Kerstin Dehne[1], Michael J Lukason[3], Akiko Noma[2], Cindy Park-Windhol[2], Mariacarmela Allocca[2], Albena Kantardzhieva[2], Shailendra Sane[2], Karolina Kosakowska[2], Brian Cafferty[2], Jan Tebbe[1], Sarah J Spencer[3], Scott Munzer[2], Christopher J. Cheng[2], Abraham Scaria[2], Andrew M. Scharenberg[2], André Cohnen[1*] and Wayne M. Coco[1*]

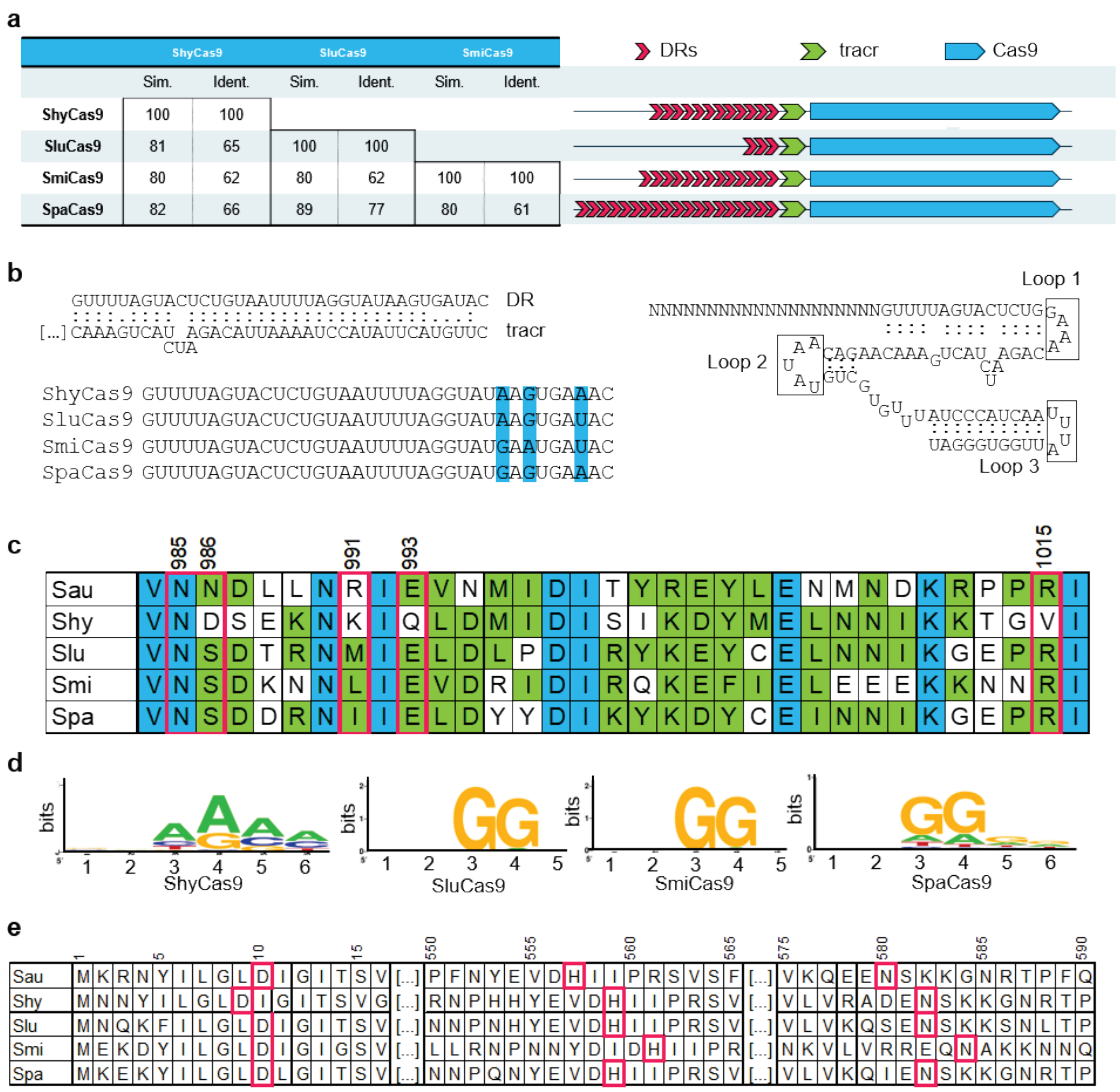

Supplementary Figure 1. Selection of four small, novel Staphylococcus Cas9s.

(a) Relatedness and genomic context of four uncharacterized *Staphylococcus* Cas9 genes: *S. hyicus* (Shy), *S. lugdunensis* (Slu), *S. microti* (Smi) and *S. pasteuri* (Spa). Each Cas9 locus includes several direct repeats (DRs) and a tracr sequence upstream of the nuclease. Loci not drawn to scale. (b) Sequences and structure of RNA components. The associated elements shared 98.8 % (tracrs) and 97.2 % (DRs) sequence identity with the corresponding SauCas9 sequences[1]. Top left: Complementarity between Slu genome locus DRs and tracr sequence. Bottom left: Alignment of the four DR sequences. Blue box: non-identities. Right: sgRNA design for SluCas9 with GAAA tetraloop (Loop 1) fusing DR and tracr[2]. Secondary structure predictions indicated similar folding for the four tracrRNAs. (c) Alignment of known and putative PAM interacting motifs in the selected *Staphylococcus* Cas9s suggests PAM-interacting residues. Amino acid numbering based on SauCas9. Strictly conserved amino acids are in

blue, chemically similar amino acids are green. Red boxes correspond to residues responsible for PAM recognition in SauCas9[3]. (d) PAM sequences as web logos. PAMs of the Cas9s were identified by in vitro-cleavage assays on a heptanucleotide (N_7) DNA library 3' of *VEGFA*_T2[4] and confirmed by bacterial survival assays carrying the same libraries. In contrast to SauCas9, 3 of the 4 chosen Cas9s recognized the short, non-degenerate 5'-NNGG-3' PAM. PAM numbering begins with the first position after the last 3' guide nucleotide. Source data are provided in the source data file. (e) Alignment to SauCas9 nuclease active site residues. Corresponding amino acids for each orthologue are highlighted with red boxes.

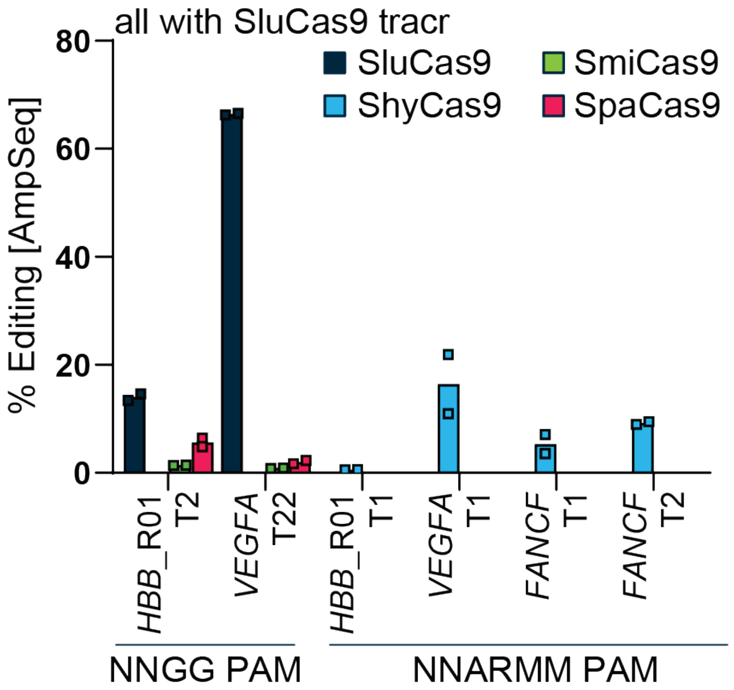

Supplementary Figure 2. **Genome editing with four Cas9s in HEK293T cells assayed by amplicon sequencing.** Shy, Smi and Slu Cas9 activity on endogenous loci in HEK293T cells using SluCas9 tracrRNA. Spa, Smi and Slu were tested for 2 targets with 5'-NNGG-3' PAM (guide_87 and guide_102 targeting the *HBB*_R01_T2 and *VEGFA*_T22 loci, respectively) and Shy with 5'-NNARMM-3' PAM (guide_1-4 targeting the *HBB*_R01_T1, *VEGFA*_T1 and *FANCF*_T1 and *FANCF*_T2 loci). Cas9s were delivered as RNPs via nucleofection and editing was analyzed via amplicon sequencing (AmpSeq). For negative controls, the respective nucleases were nucleofected in absence of sgRNA. Editing values were normalized against background, n = 2 independent biological replicates, source data are provided in the source data file.

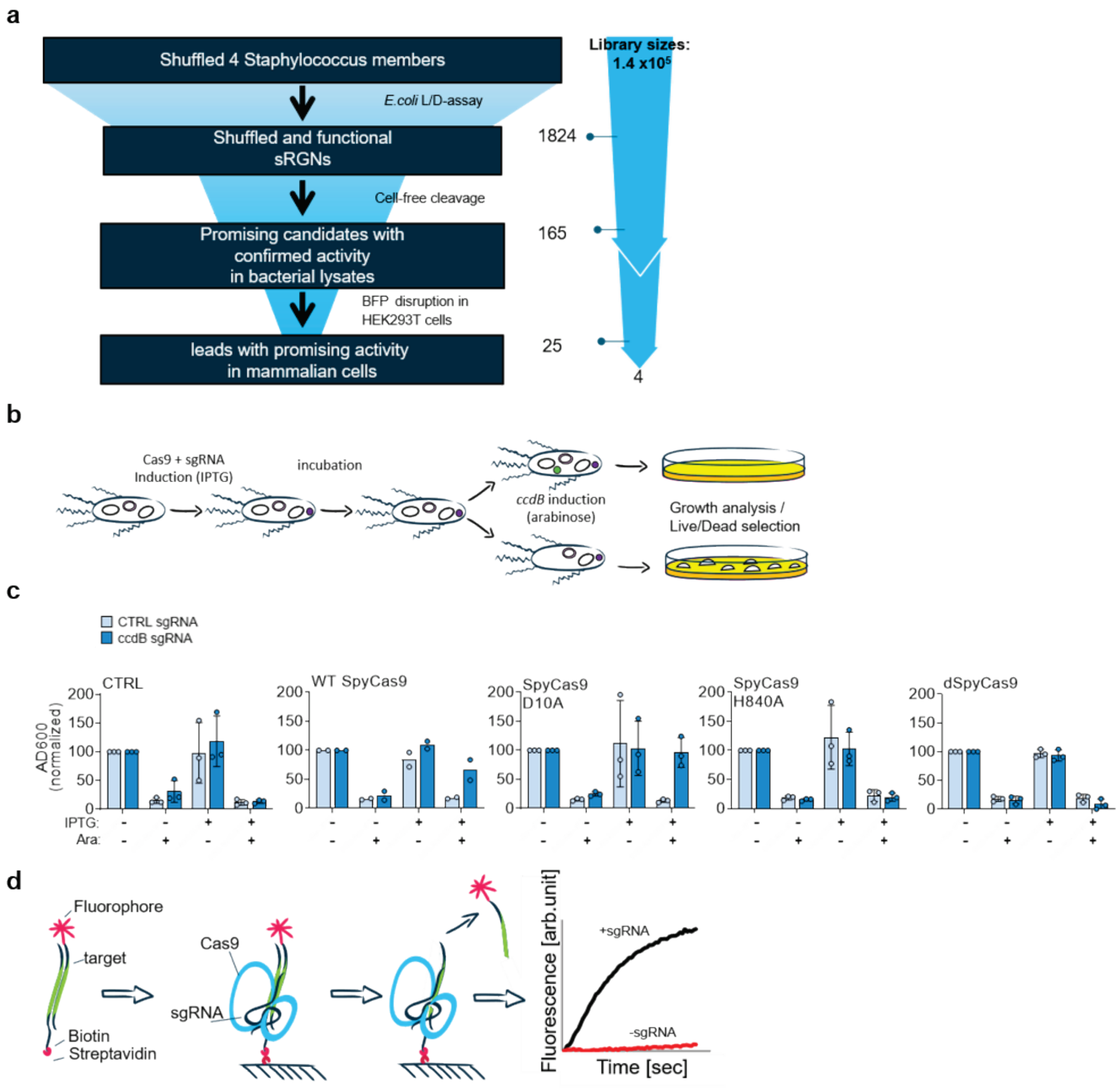

Supplementary Figure 3. Screening assays for functional sRGN variants. (a) Screening approach used to identify improved sRGNs by protein engineering. The succession of screening assays is shown on the left with the corresponding number of sRGN variants that progressed on the right. 25 sRGN variants were identified as top hits in the final BFP disruption screen in HEK293T cells[5]. (b) Schematic for “live/dead” (L/D) bacterial survival assay. Cells were generated harbouring an arabinose-inducible toxic *ccdB* gene reporter plasmid, a second plasmid harbouring a transcription cassette for the corresponding sgRNA, and a third plasmid encoding an IPTG-inducible, Trc promoter-controlled nuclease gene. Active Cas9/sgRNA complexes successfully cleave the toxic reporter, which is inactivated by cleavage at the *VEGFA*-T2 target site (guide_113), and cells survive under selection conditions. Open circles are cartoons of plasmids, green circle depicts *ccdB* gene product, purple circle represents nuclease. (c) L/D assay validation using SpyCas9 and SpyCas9 mutants. WT = SpyCas9; D10A and H840A = nickases; and dSpyCas9 = catalytically inactive SpyCas9. Upon both

arabinose and IPTG induction, SpyCas9- or D10A-expressing cells survive while those that express H840A or dSpyCas9 do not, WTSpyCas9 n = 2 independent biological replicates, all other data n = 3 independent biological replicates, data are presented as mean ± SD. Source data are provided in the source data file. (d) Fluorescence polarization assay (FP Assay) schematic. Biotinylated and ATTO647N-labelled oligonucleotide duplexes were immobilized on streptavidin coated plates. RNP complexes were formed, and cleavage of the dsDNA was monitored by following decreasing anisotropy and increasing fluorescence intensity, arb.unit = arbitrary units.

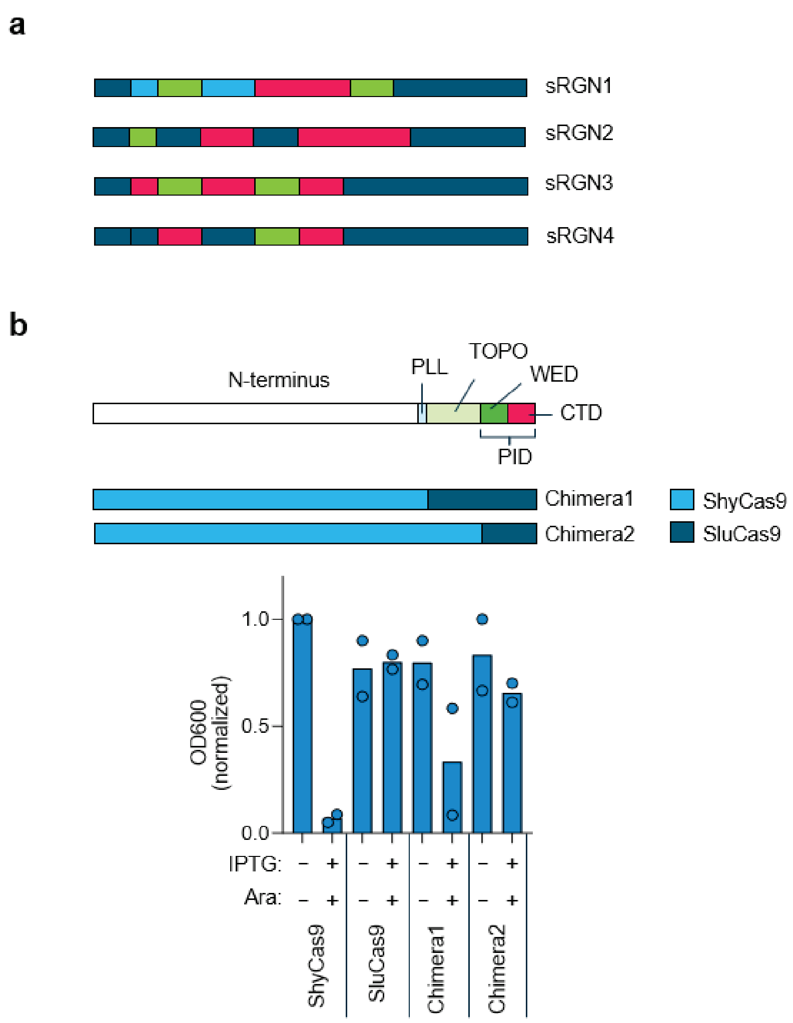

Supplementary Figure 4. Rational exchange of PAM-interacting domains. (a) Shuffling fragment architecture of screening hits sRGN1, sRGN2, sRGN3 and sRGN4. Dark blue = SluCas9 segments, light blue = ShyCas9 segments, green = SmiCas9 segments, pink = SpaCas9 segments. (b) Rationally swapping PI-domains (PID) and WEDGE/PI-domains from the NNGG-recognizing SluCas9 to ShyCas9 alters PAM motif recognition and creates functionally active proteins. Chimera 1 and 2 substitute SluCas9 WEDGE/PI domain amino acids 739-1053 and 910-1053, respectively, into the ShyCas9 gene. Bacterial live/dead growth analysis on the *VEGFA*_T2 target sequence (guide_113) with a NNGG-PAM, indicates that both chimeric constructs gained the ability to effectively employ an NNGG PAM motif, while the unaltered ShyCas9, as expected, cannot. Data presented is the mean of n = 2 independent biological replicates, source data are provided in the source data file. PLL = phosphate lock loop, CTD = C-terminal domain, WED = WEDGE domain, TOPO = topoisomerase domain, Ara = arabinose.

a

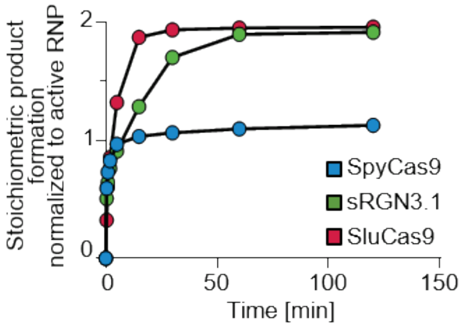

b

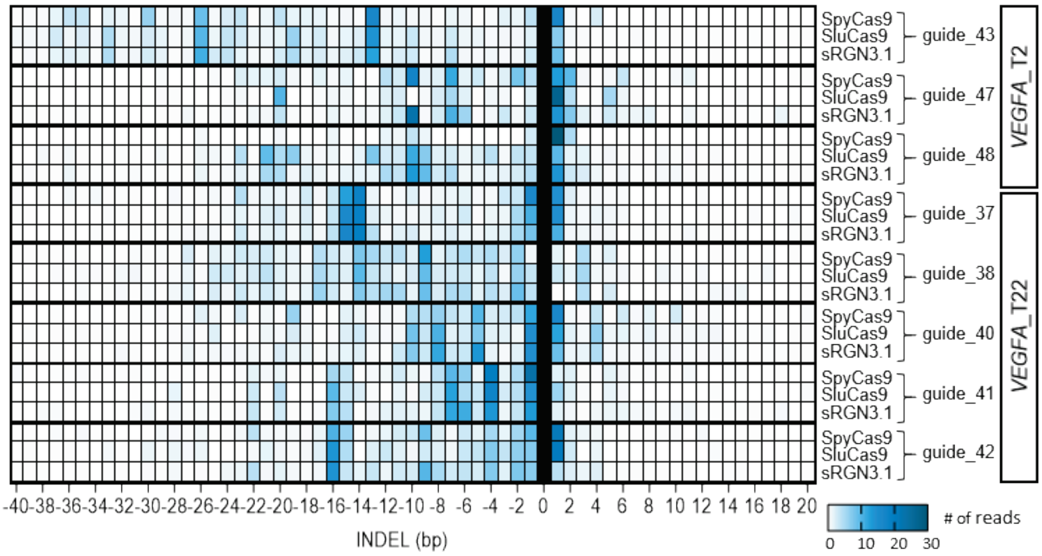

Supplementary Figure 5. Catalytic turnover and indel patterns. (a) Plasmid containing the on-target sequence, 5'-TCGTAAAGTGGTGCGTTCTC-3', was mixed with the indicated nuclease at a DNA:RNP molar ratio of 2:1. At the indicated times, reactions were quenched and analyzed, data presented are n = 1 biological replicate. Stoichiometric product formation ratios greater than 1 indicate that single nuclease molecules cleaved multiple substrate molecules. (b) Insertion and deletion (indel) pattern for SpyCas9, SluCas9 and sRGN3.1 on eight target sites within the *VEGFA* locus (for guide sequences, see Supplementary Table 1). Amplicon sequencing was performed and indel identity was calculated by CRISPResso. Data are presented as mean of n = 2-3 independent biological replicates, except Spy (guide_37) n = 1 independent biological replicate, source data are provided in the source data file. Unaltered reads (indel = 0 bp, black squares) were excluded from the analysis.

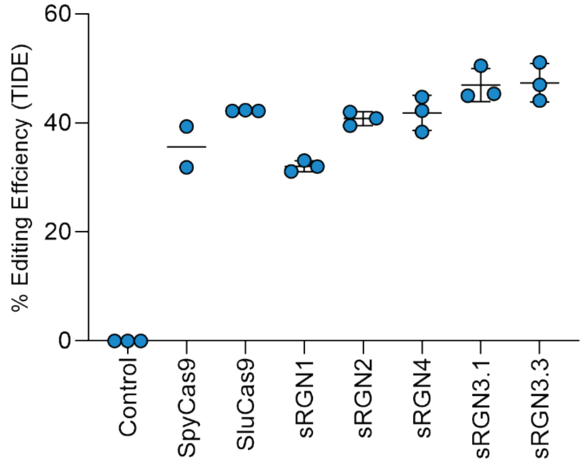

Supplementary Figure 6. Genome editing in a murine hepatoma cell line. Genome editing performance of SpyCas9, SluCas9, sRGN3.1, sRGN3.3, sRGN1, sRGN2, and sRGN4 variants in murine cells was assessed by transfection of Hepa1-6 cells with the respective albumin locus-targeting mRNAs (see Supplementary Table 2) and sgRNAs (*Alb*-T1 target, guide 112, see Supplementary Table 1 for sequence), n = 3 independent biological replicates for sRGNs and SluCas9, data are presented as mean ± SD, and n = 2 for SpyCas9). Source data are provided in the source data file.

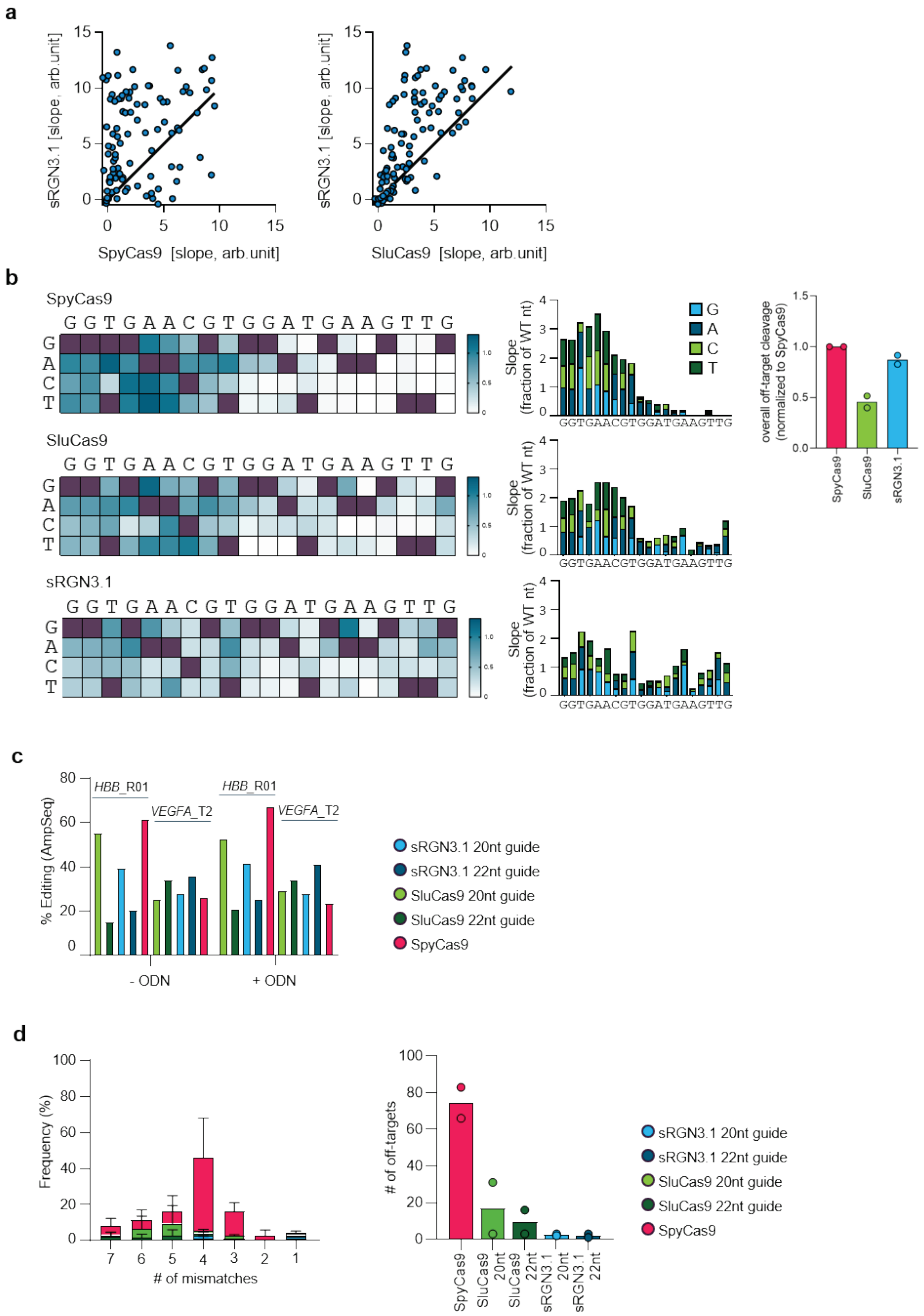
a
sRGN3.1 [slope, arb.unit]
SpyCas9 [slope, arb.unit]
SluCas9 [slope, arb.unit]
0
5
10
15
b
SpyCas9
SluCas9
sRGN3.1
G G T G A A C G T G G A T G A A G T T G
G
A
C
T
Slope (fraction of WT nt)
GGTGAACGTGGATGAAGTTG
overall off-target cleavage (normalized to SpyCas9)
0.0
0.5
1.0
1.5
c
% Editing (AmpSeq)
HBB_R01
VEGFA_T2
- ODN
+ ODN
sRGN3.1 20nt guide
sRGN3.1 22nt guide
SluCas9 20nt guide
SluCas9 22nt guide
SpyCas9
d
Frequency (%)
# of mismatches
7
6
5
4
3
2
1
# of off-targets
SpyCas9
SluCas9 20nt
SluCas9 22nt
sRGN3.1 20nt
sRGN3.1 22nt
sRGN3.1 20nt guide
sRGN3.1 22nt guide
SluCas9 20nt guide
SluCas9 22nt guide
SpyCas9

Supplementary Figure 7. Extended activity and specificity assessments. (a) Activity comparison plots of sRGN3.1 vs. SpyCas9 and SluCas9 on 96 targets. 48 therapeutically relevant targets and 48 rationally designed targets (engineered to explore GC-content between 20% and 80 %, see Supplementary Table 1 for sequences) were probed with SpyCas9, SluCas9 and sRGN3.1 in the FP cleavage assay. Each dot depicts the mean initial slope for each reaction, arb.unit = arbitrary units. Line visualizes x = y; data are presented in mean and are n = 2 independent biological replicates. (b) Left: Nuclease specificity assessment by cell-free FP assay at all possible single nucleotide target::gRNA mismatches. Heatmap ranges from white (no off-target cleavage) to dark blue (extensive off-target cleavage), purple box = WT nucleotide. PAM (not depicted) is to the right of the displayed sequences. Middle: alternative depiction of these data with relative initial rate values (slope) for each mismatch position, colors indicate the respective mutation at each position. Overall specificity for SpyCas9 and SluCas9 was about equal, while overall specificity of sRGN3.1 was 15% higher than SpyCas9. Right: on-target cleavage in these experiments relative to SpyCas9, data are presented as mean with n = 2 independent biological replicates. (c) On-target editing observed with the nuclease concentrations selected by titration as input for the GUIDE-Seq experiment, with or without double-stranded oligodeoxynucleotide (dsODN), required for capturing of off-targets in GUIDE-Seq experiments. 60 pmol SpyCas9 and 30 pmol each of SluCas9 and sRGN3.1 were used for targeting *HBB*-R01, except for sRGN3.1 with 22nt guide, 8pmol were used. For targeting *VEGFA*_T2, 60 pmol SpyCas9, 8 and 18 pmol sRGN3.1 (for 20nt and 22nt guide, respectively) and 6 and 10 pmol SluCas9 (for 20nt and 22nt guide, respectively), n = 1. (d) Quantification of off-targets retrieved for each nuclease by GUIDE-Seq by number of mismatches (left) or overall number of off-targets (right). Results for both targets (*HBB*_R01 and *VEGFA*_T2) were combined for these plots. Source data for all subfigures are provided in the source data file.

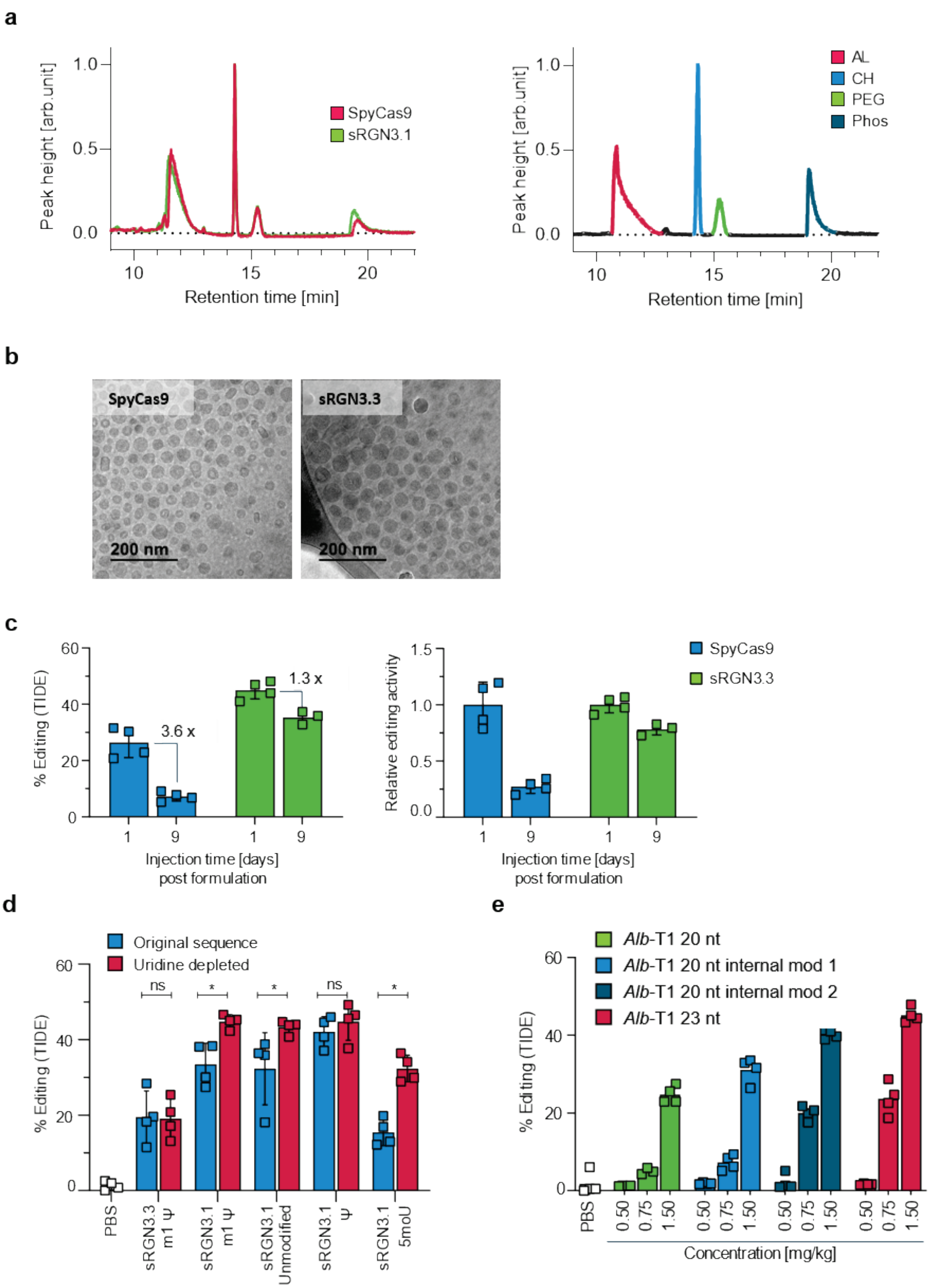

Supplementary Figure 8. LNP-mediated in vivo editing of sRGNs. (a) UPLC analysis of sRGN3.1 and SpyCas9 LNPs. Left: Representative chromatogram. sRGN3.1 mRNA LNPs had a size of 74 nm, polydispersity index (PDI) of 0.11, and RNA entrapment of 96%; SpyCas9 mRNA LNPs had a size of 74 nm, PDI of 0.13, and RNA entrapment of 91%. Lipid standards (right) were analyzed to identify peaks. sRGN3.1 LNPs showed a slight reduction in amino lipid

(AL) and an increase in 1,2-dioleoyl-sn-glycero-3-phosphoethanolamine (18:1(Δ9-Cis)PE (DOPE) phospho-lipid (Phos) content compared to SpyCas9 LNPs, suggesting altered lipid-RNA associations compared to SpyCas9 mRNA, arb.unit = arbitrary units, CH = cholesterol, PEG = poly-ethylene-glycol, black line = background. (b) CryoTEM morphology analysis of sRGN3.3 and SpyCas9 LNPs. sRGN LNPs displayed improved circularity and multilamellar structure. One representative image of preparations shown in 8 c. (c) SpyCas9-LNP and sRGN3.3-LNP functional stability at 4°C. Liver editing in mice was assessed on indicated days upon intravenous administration. Left: dose of 2 mg/kg, n = 4 independent biological replicates, mean ± S.D. Right: Normalization of the data to day 1 showed 3.6-fold activity reduction for SpyCas9-LNPs and 1.3-fold activity reduction for sRGN3.3-LNPs. (d) Functional in vivo evaluation via TIDE analysis of uridine depletion for sRGN3.1 and sRGN3.3 mRNA constructs with different uridine-substituted base modifications. sRGN3.3 mRNA with (N1)-methylpseudouridine (m1Ψ) or sRGN3.1 with pseudouridine (Ψ) showed no significant difference with uridine depletion; whereas sRGN3.1 mRNA with m1Ψ, 5-methoxyuridine (5moU), and no modification showed significantly increased editing with uridine depletion, (dose of 1 mg/kg, n = 4 independent biological replicates, mean ± SD). For all other in vivo studies m1Ψ modification and the non-uridine depleted constructs were used. Significance was determined using the Mann Whitney test, (*) = $p < 0.05$, ns = not significant. (e) In vivo evaluation of sgRNA modification approaches in mice via TIDE analysis. Tested were internal chemical modifications (Internal mod 1 and 2) and increasing protospacer length from 20 to 23 nt (Supplementary Table 1). Liver editing showed a dose response at 0.5, 0.75, and 1.5 mg/kg of total LNP-encapsulated RNA. Both modification strategies showed improved potency compared to standard modified sgRNAs. Protospacer length of 23 nt (standard modifications) showed highest potency. N = 4 independent biological replicates, mean ± SD. Source data are provided in the source data file.

a

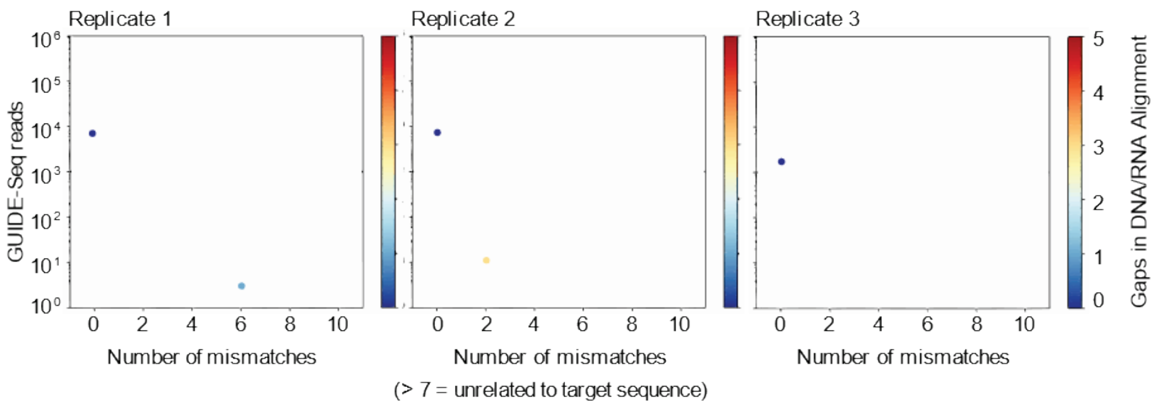

b

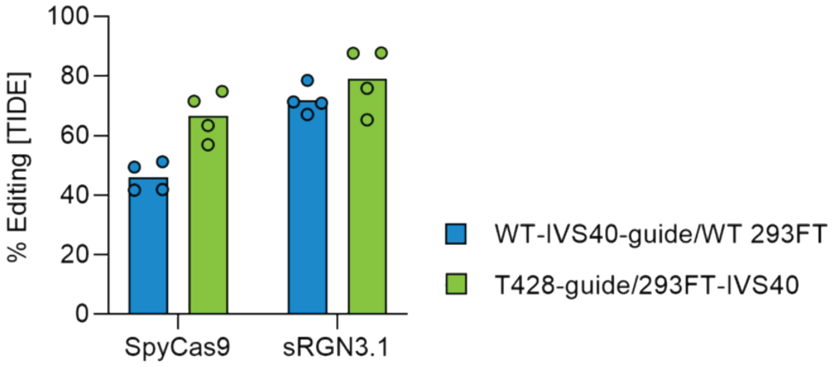

Supplementary Figure 9. **Editing at the intron 40 of the *USH2A* gene locus.** (a) The 7595-2144A > G Usher disease mutation in intron 40 of *USH2A* (IVS40) results in an additional exon being incorporated in the *USH2A* mRNA. The T429 IVS50 guide (guide_110) targets the mutant IVS40 allele, while WT-IVS40 targets the WT (Supplementary Table 1, guide_111). GUIDE-seq revealed no sRGN off-target sites above background for T428-IVS40-guide and confirmed via AmpSeq (source data file, Supplementary Fig. 9). A plasmid carrying the sRGN3.1 gene and T428-IVS40-guide was nucleofected into the homozygous IVS40 293FT (293FT-IVS40) cell line together with dsODN. Break sites in which dsODN was inserted were identified by NGS. Only genomic sequences quite distant (> 7 mismatches and multiple alignment gaps) from the on-target site for T428-IVS40-guide were captured by dsODN at relevant read counts, suggesting that these sites were not cleaved as off-targets by sRGN3.1 complexed with T428-IVS40-guide. Background was 0.26 % for SpyCas9 and 0.07 % for sRGN3.1. Three replicates for sRGN3.1are shown, identical scales for each replicate. (b) Comparison of editing with mutant IVS40 allele targeting guide and its surrogate guide. 293FT-IVS40 cell line and its WT parent cell line were transfected with a plasmid carrying either SpyCas9 or sRGN3.1 and sgRNA that matched either WT (guide_111) or the IVS40 SNP allele

(guide_110). WT-IVS40-guide differs from T428-IVS40-guide by a single nucleotide and completely matches the wild type *USH2A* intronic sequence of NHP and human. Insertions and deletions (indels) were quantified using cells harvested 7 days after transfection via TIDE analysis, n = 2 biologically independent experiments, 2 technical replicas each, each datapoint is shown. Source data are provided in the source data file.

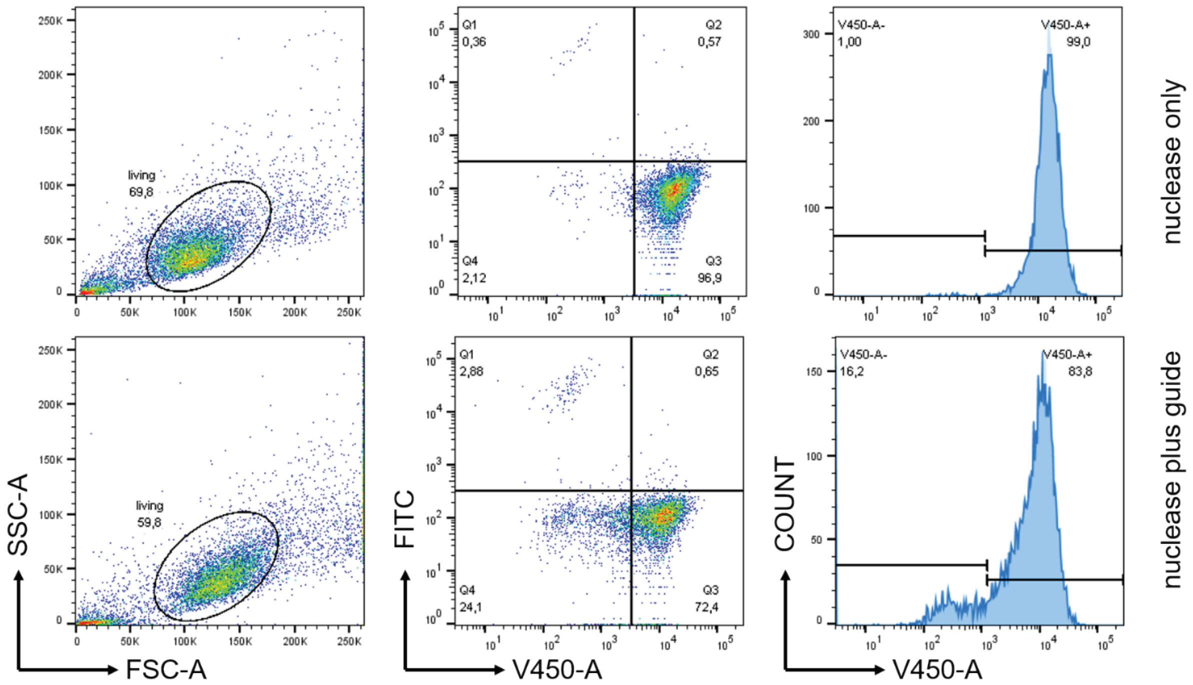

Supplementary Figure 10. Gating strategy for FACS analysis of BFP-disruption. HEK293T cells, harboring a *BFP* cassette in the *AAVS*1 locus were transfected with nuclease expression plasmid (with T2A-GFP) and guide expression plasmid (guide 5-9 and 11-16 for sRGNs and guide_19-23 and 25-30 for SpyCas9). Cells were FACS-analyzed 7 days post transfection. Shown is the gating strategy for the control (nuclease only) and a nuclease plus guide treated sample, as used for evaluation of % BFP disruption as presented in Figure 2. A signal from minimum of 10,000 cells was assayed using the V450 filter set in the BD FACS canto II and software diva 8.0.1 and FlowJo 10.7.2.

Supplementary References

1. Ran, F. A. *et al.* In vivo genome editing using Staphylococcus aureus Cas9. *Nature* **520**, 186–191 (2015).
2. Jinek, M. *et al.* A programmable dual-RNA-guided DNA endonuclease in adaptive bacterial immunity. *Science* **337**, 816–821 (2012).
3. Nishimasu, H. *et al.* Crystal Structure of Staphylococcus aureus Cas9. *Cell* **162**, 1113–1126 (2015).
4. Fu, Y. *et al.* High-frequency off-target mutagenesis induced by CRISPR-Cas nucleases in human cells. *Nature Biotechnology* **31**, 822–826 (2013).
5. Kleinstiver, B. P. *et al.* Engineered CRISPR-Cas9 nucleases with altered PAM specificities. *Nature* **523**, 481–485 (2015).
