# Supplementary material for: Improved CRISPR genome editing using small highly active and specific engineered RNA-guided nucleases
Source: Nat Commun. 2021 Jul 9;12:4219. doi: 10.1038/s41467-021-24454-5 (PMC8271026; doi:10.1038/s41467-021-24454-5)
Supplement: Supplementary file 5 — Supplementary Data 4 [file 41467_2021_24454_MOESM5_ESM.zip › 293708_1_data_set_5645160_qfhf7h (1).docx]

On-target and off-target editing evaluation of sRGN3.1 on the IVS40 mutation

Forty-nine sites containing up to 5 nucleotide substitutions from the T428 target site were selected for this analysis. The homozygous IVS40 293FT cell line or its parental cell line 293FT were transfected with T428 sgRNA by Lipofectamine 3000. Genomic DNA was extracted 7 days after nucleofection and INDELs were analyzed by amplicon sequencing. Fasta files correspond to data provided in the source data file (Supplementary Figure 9a).
