# Supplementary material for: Improved CRISPR genome editing using small highly active and specific engineered RNA-guided nucleases
Source: Nat Commun. 2021 Jul 9;12:4219. doi: 10.1038/s41467-021-24454-5 (PMC8271026; doi:10.1038/s41467-021-24454-5)
Supplement: Supplementary file 6 — Supplementary Software [file 41467_2021_24454_MOESM6_ESM.zip › NCOMMS-21-01888A ZIP/293708_1_supp_5638210_qpfp13.docx]

Assumption 1: the PAM-construct (i.e. on a plasmid) looks like the following

-constant_seq1 – target_seq-PAMLIB – constant_seq2

Where target_seq is the sequence targeted by the gRNA, PAMLIB is a stretch of 7-8 degenerate (N) nucleotides, and constant_seq1 and constant_seq2 are constant sequences up- and downstream of the target / pam region

Assumption 2: the NGS runs of the required samples (i.e. treated/nuclease+gRNA and mock) have been properly processed, and one trimmed/joined/demultiplexed .fastq file for each sample has been obtained.

To get the potential PAM, two analysis steps / commandlines are required

1. Getting relevant reads from the sample fastq file (i.e. those that contain the site of interest)

Run the following commandline:

python src/SimplifyFastQSubsequence.py --fastq_file <your fastqfile> --sequence <motif from constant seq 1> --seq_window 30 --search_start 1

where <motif from constant seq1> should be a ~10 nucleotide stretch (that should be unique in the plasmid, or at least the amplicon) right upstream of the target seq, and that shouldn’t be touched by the nuclease

The output of this commaindline is another fastq file that only contains reads that have constant motif. For each of these reads, only the number of surrounding residues mentioned by the -seq_window option are written out.

This commandline needs to be called for every fastq file of interest with the same –sequence and –seq_window parametes

1. Getting the PAM from the simplified fastq files

2.1 for depletion, the following commandline needs to be called

python src/AnalyzeNGSVariants.py --first_rel_nt <X1> --last_rel_nt <X2> --fastq_naive <simplified mock .fastq file> --fastq_selected <simplified treated .fastq file> --min_nt_qual + --num_top_vars 100 --analysis_mode depletion > sample_depletion_out.txt

where X1 and X2 are integer numbers that specify the sequence region that the depletion analysis will be carried out on. These two numbers should be selected such that they span the rgion of the PAMLIB in the simplified file. I.e. in the above simplification example, if the sequence motif had length 10 and seqwindow was 30 (where the PAMLIB was 8N starting 20 nucleotides downstream of the sequence motif), then the sequences in the fastq file should each be 70 bases long, with the PAMLIB being from 60 to 68. Values for X1 and X2 could thus be 58 and 70

The output file will contain a list of the most depleted sequences, and at the end of the file, after lie “Top ranked ntarray (built from top 100 variants) looks like:”, also a matrix containing the nucleotide frequencies at each of the positions between X1 and X2 in the top 100 positions. The columns of the matrix are the sequence positions, the five rows are the four bases + wildcard N in order A, C, G, T. N

Any high numbers here are indicative of a PAM. For example, if the matrix is

[[ 0.19 0.24 0.23 0.18 0.27 0.31 0.28]

[ 0.11 0.12 0.1 0.16 0.13 0.13 0.13]

[ 0.41 0.56 0.53 0.27 0.28 0.35 0.32]

[ 0.29 0.08 0.14 0.39 0.32 0.21 0.27]

[ 0. 0. 0. 0. 0. 0. 0. ]]

That means that a G is over-represented at the first, second, and third position, indicative of a GGG PAM
