# Supplementary material for: Improved CRISPR genome editing using small highly active and specific engineered RNA-guided nucleases
Source: Nat Commun. 2021 Jul 9;12:4219. doi: 10.1038/s41467-021-24454-5 (PMC8271026; doi:10.1038/s41467-021-24454-5)
Supplement: Supplementary file 7 — Reporting Summary [file 41467_2021_24454_MOESM7_ESM.pdf]

nature research

Corresponding author(s): André Cohnen, Wayne M Coco

Last updated by author(s): May 20, 2021

Reporting Summary

Nature Research wishes to improve the reproducibility of the work that we publish. This form provides structure for consistency and transparency in reporting. For further information on Nature Research policies, see our Editorial Policies and the Editorial Policy Checklist.

Statistics

For all statistical analyses, confirm that the following items are present in the figure legend, table legend, main text, or Methods section.

n/a	Confirmed	
☐	☒	The exact sample size (*n*) for each experimental group/condition, given as a discrete number and unit of measurement
☐	☒	A statement on whether measurements were taken from distinct samples or whether the same sample was measured repeatedly
☐	☒	The statistical test(s) used AND whether they are one- or two-sided<br>*Only common tests should be described solely by name; describe more complex techniques in the Methods section.*
☒	☐	A description of all covariates tested
☐	☒	A description of any assumptions or corrections, such as tests of normality and adjustment for multiple comparisons
☐	☒	A full description of the statistical parameters including central tendency (e.g. means) or other basic estimates (e.g. regression coefficient) AND variation (e.g. standard deviation) or associated estimates of uncertainty (e.g. confidence intervals)
☐	☒	For null hypothesis testing, the test statistic (e.g. *F*, *t*, *r*) with confidence intervals, effect sizes, degrees of freedom and *P* value noted<br>*Give P values as exact values whenever suitable.*
☒	☐	For Bayesian analysis, information on the choice of priors and Markov chain Monte Carlo settings
☒	☐	For hierarchical and complex designs, identification of the appropriate level for tests and full reporting of outcomes
☐	☒	Estimates of effect sizes (e.g. Cohen's *d*, Pearson's *r*), indicating how they were calculated

Our web collection on statistics for biologists contains articles on many of the points above.

Software and code

Policy information about availability of computer code

Data collection	Illumina MiSeq software (version 2.6.2.1) was used on the Illumina MiSeq sequencers to collect NGS data
Data analysis	GUIDE-seq experiments were analyzed using: https://github.com/aryeelab/guideseq commit version c608522P.<br>TIDE experiments were analyzed using TIDE as published in doi:10.1093/nar/gku936 (2014).<br>Individual NGS samples were analyzed using CRISPResso (version V 1.0.13), as published in doi:10.1089/nat.2017.0697 (2018).<br>PAM-ID experiements were analyzed via custom code and is available upon request.<br>Demultiplexing was performed using a custom code and is available upon request.<br>Python program version 2.7 was used for coding.

For manuscripts utilizing custom algorithms or software that are central to the research but not yet described in published literature, software must be made available to editors and reviewers. We strongly encourage code deposition in a community repository (e.g. GitHub). See the Nature Research guidelines for submitting code & software for further information.

Data

Policy information about availability of data

All manuscripts must include a data availability statement. This statement should provide the following information, where applicable:

- Accession codes, unique identifiers, or web links for publicly available datasets
- A list of figures that have associated raw data
- A description of any restrictions on data availability

All data generated or analyzed during this study are included in this published article and its supplementary information files and are available from the corresponding author upon reasonable request. Parental Staphylococcus sequences can be accessed on Uniprot: S.hyicus (Shy) GB: CP008747.1, Uniprot:

A0A418JLD8, S.lugdunensis (Slu) NCBI reference sequence: NZ_GL622352.1, Uniprot: A0A133QCR3,S.microti(Smi) GB: JXWY01000132.1, Uniprot:A0A0D6XNZ8 and S.pasteuri (Spa) GB: CP004014.1. Sequences of engineered proteins used herein are available in Supplementary Table 2. Source data are provided with this paper. NGS raw data are provided through Sequence Read Archive (SRA), project ID PRJNA731307.

Field-specific reporting

Please select the one below that is the best fit for your research. If you are not sure, read the appropriate sections before making your selection.

☒ Life sciences ☐ Behavioural & social sciences ☐ Ecological, evolutionary & environmental sciences

For a reference copy of the document with all sections, see nature.com/documents/nr-reporting-summary-flat.pdf

Life sciences study design

All studies must disclose on these points even when the disclosure is negative.

Sample size	Sample size and statistic test for each experiment is provided in the respective figure legend. In general, sample sizes were chosen to allow the statistical determination of differences between the sets of quantities compared for by the statistical measures that were used.
Data exclusions	No data was excluded
Replication	Excepting the high-throughput screening of library variants (were replication is not standard practice, see above), all main-figure experiments were repeated / reproduced as stated in the figure legend. GUIDE-Seq experiments were conducted once per guide but the resulting, reproducible rankings/trends with two different guides served as a mutual confirmation of the reliability of each set.
Randomization	Cells (both bacteria and mammalian cells) used in this study were grown under identical conditions. Randomization was not used.
Blinding	Samples were prepared unblinded but in parallel. Analysis was performed based on numerical sample names, without the identity of the samples known during the analysis. Cells (both bacteria and mammalian cells) used in this study were grown under identical conditions.

Reporting for specific materials, systems and methods

We require information from authors about some types of materials, experimental systems and methods used in many studies. Here, indicate whether each material, system or method listed is relevant to your study. If you are not sure if a list item applies to your research, read the appropriate section before selecting a response.

Materials & experimental systems

n/a	Involved in the study	
☒	☐	Antibodies
☐	☒	Eukaryotic cell lines
☒	☐	Palaeontology and archaeology
☐	☒	Animals and other organisms
☒	☐	Human research participants
☒	☐	Clinical data
☒	☐	Dual use research of concern

Methods

n/a	Involved in the study	
☒	☐	ChIP-seq
☐	☒	Flow cytometry
☒	☐	MRI-based neuroimaging

Eukaryotic cell lines

Policy information about cell lines

Cell line source(s)	HEK293T (ref. CRL-3216, ATCC), murine Hepa 1-6 (ref. CRL-1830, ATCC) and 293FT cells (ref. R70007, Thermo Fisher Scientific)
Authentication	Cells were authenticated by the supplier via certificate of analysis.
Mycoplasma contamination	All cell lines tested negative for mycoplasma.
Commonly misidentified lines (See ICLAC register)	No commonly misidentified cell lines were used in the study.

Animals and other organisms

Policy information about studies involving animals; ARRIVE guidelines recommended for reporting animal research

Laboratory animals	Macaca fascicularis, Age: 2 to 4 years old, Weight: 2.5 to 5.0 kg, Sex: Male, Source: Bred in captivit at World Wide Primates, Inc., Origin: Mainland Asia

	C57BL/6j mice, Age: 6 to 8 weeks old, Sex: Male, Source: Bred in captivity at Jackson Laboratories, Bar Harbor, Maine, Origin: Jackson Laboratories, Bar Harbor, Maine
Wild animals	This study did not involve wild animals.
Field-collected samples	This study did not involve samples collected from the field.
Ethics oversight	This study complied with all applicable sections of the Final Rules of the Animal Welfare Act regulations (Code of Federal Regulations, Title 9), the Public Health Service Policy on Humane Care and Use of Laboratory Animals from the Office of Laboratory Animal Welfare, and the Guide for the Care and Use of Laboratory Animals from the National Research Council. The protocol and any amendments or procedures involving the care or use of animals in this study was reviewed and approved by the Testing Facility Institutional Animal Care and Use Committee before the initiation of such procedures.<br><br>Testing Facility Institutional Animal Care and Use Committee: Charles River Laboratories, Mattawan, Michigan 49071 (Macaca fascicularis) and Mispro Biotech Services, Inc. (C57BL/6j)

Note that full information on the approval of the study protocol must also be provided in the manuscript.

Flow Cytometry

Plots

Confirm that:

- [x] The axis labels state the marker and fluorochrome used (e.g. CD4-FITC).
- [x] The axis scales are clearly visible. Include numbers along axes only for bottom left plot of group (a 'group' is an analysis of identical markers).
- [x] All plots are contour plots with outliers or pseudocolor plots.
- [x] A numerical value for number of cells or percentage (with statistics) is provided.

Methodology

Sample preparation	HEK293T were prepared by washing with 1X PBS, trypsinization, and resuspension in 200 µl FACS buffer (1X PBS supplemented with 2% FCS).
Instrument	BD FACS Canto II
Software	BD FACS DIVA 8.0.1 and FlowJo 10.7.2.
Cell population abundance	no cell sorting was conducted in this study, a minimum of 10k cells were analyzed per sample analyzed
Gating strategy	FSC-A/SSC-A followed by V450-A/ FITC-A. Gates were set as quadrants left of V450 (BFP) positive cells and cells shifting from Q4 to Q3 were counted as BFP negative

- [x] Tick this box to confirm that a figure exemplifying the gating strategy is provided in the Supplementary Information.
