# Supplementary material for: Improved CRISPR genome editing using small highly active and specific engineered RNA-guided nucleases
Source: Nat Commun. 2021 Jul 9;12:4219. doi: 10.1038/s41467-021-24454-5 (PMC8271026; doi:10.1038/s41467-021-24454-5)
Supplement: Supplementary file 8 — Description of Additional Supplementary Files [file 41467_2021_24454_MOESM8_ESM.pdf]

Additional Supplementary Files

Title: Supplementary Data 1

Description: Overview of guides, tracr and repeat sequences used in this study.

Title: Supplementary Data 2

Description: Sequences of nucleases used in this study.

Title: Supplementary Data 3

Description: Overview of primers, probes and oligos used in this study.

Title: Supplementary Data 4

Description: Data supplementing off-target analysis in HEK293FT cells. On-target and off-target editing evaluation of sRGN3.1 on the IVS40 mutation. Forty-nine sites containing up to 5 nucleotide substitutions from the T428 target site were selected for this analysis. The homozygous IVS40 293FT cell line or its parental cell line 293FT were transfected with T428 sgRNA by Lipofectamine 3000. Genomic DNA was extracted 7 days after nucleofection and INDELs were analyzed by amplicon sequencing. Fasta files correspond to data provided in the source data file (Supplementary Figure 9a).

Title: Supplementary Software

Description: Code for PAM analysis used in this study; Code for demultiplexing used in this study.

RESTRICTED
