# Supplementary material for: Improved CRISPR genome editing using small highly active and specific engineered RNA-guided nucleases
Source: Nat Commun. 2021 Jul 9;12:4219. doi: 10.1038/s41467-021-24454-5 (PMC8271026; doi:10.1038/s41467-021-24454-5)
Supplement: Supplementary file 9 — Peer Review File [file 41467_2021_24454_MOESM9_ESM.pdf]

Editorial Note: This manuscript has been previously reviewed at another journal that is not operating a transparent peer review scheme. This document only contains reviewer comments and rebuttal letters for versions considered at *Nature Communications*. Mentions of prior referee reports have been redacted.

Reviewers' Comments:

Reviewer #2:
Remarks to the Author:
The authors have addressed all the major issues I raised in their revised manuscript.
